# Genetic and lipidomic analyses suggest that *Nostoc punctiforme*, a plant-symbiotic cyanobacterium, does not produce sphingolipids

**DOI:** 10.1099/acmi.0.000306

**Published:** 2022-01-21

**Authors:** Samuel Belton, Nadia Lamari, Lars S. Jermiin, Vicente Mariscal, Enrique Flores, Paul F. McCabe, Carl K. Y. Ng

**Affiliations:** ^1^​ UCD School of Biology and Environmental Science, University College Dublin, Belfield, Dublin D4, Ireland; ^2^​ UCD Earth Institute, O’Brien Centre for Science, University College Dublin, Belfield, Dublin D4, Ireland; ^3^​ Research School of Biology, Australian National University, Canberra, ACT 2600, Australia; ^4^​ Instituto de Bioquímica Vegetal y Fotosíntesis, Consejo Superior de Investigaciones Científicas and Universidad de Sevilla, cicCartuja, Avda. Américo Vespucio 49, 41092 Seville, Spain; ^5^​ UCD Centre for Plant Science, University College Dublin, Belfield, Dublin D4, Ireland; ^†^​ Present address: DBN Plant Molecular Biology Lab, National Botanic Gardens of Ireland, Dublin, Ireland; ^‡^​ Present address: Philip Morris International, Quai Jeanrenaud 3, 2000, Neuchâtel, Switzerland

**Keywords:** sphingolipids, long chain base, serine palmitoyltransferase, *Nostoc*, cyanobacteria, symbiosis

## Abstract

Sphingolipids, a class of amino-alcohol-based lipids, are well characterized in eukaryotes and in some anaerobic bacteria. However, the only sphingolipids so far identified in cyanobacteria are two ceramides (i.e.*,* an acetylsphingomyelin and a cerebroside), both based on unbranched, long-chain base (LCB) sphingolipids in *Scytonema julianum* and *Moorea producens *, respectively. The first step in *de novo* sphingolipid biosynthesis is the condensation of l-serine with palmitoyl-CoA to produce 3-keto-diyhydrosphingosine (KDS). This reaction is catalyzed by serine palmitoyltransferase (SPT), which belongs to a small family of pyridoxal phosphate-dependent α-oxoamine synthase (AOS) enzymes. Based on sequence similarity to molecularly characterized bacterial SPT peptides, we identified a putative SPT (Npun_R3567) from the model nitrogen-fixing, plant-symbiotic cyanobacterium, *Nostoc punctiforme* strain PCC 73102 (ATCC 29133). Gene expression analysis revealed that *Npun_R3567* is induced during late-stage diazotrophic growth in *N. punctiforme *. However, Npun_R3567 could not produce the SPT reaction product, 3-keto-diyhydrosphingosine (KDS), when heterologously expressed in *Escherichia coli *. This agreed with a sphingolipidomic analysis of *N. punctiforme* cells, which revealed that no LCBs or ceramides were present. To gain a better understanding of Npun_R3567, we inferred the phylogenetic position of Npun_R3567 relative to other bacterial AOS peptides. Rather than clustering with other bacterial SPTs, Npun_R3567 and the other cyanobacterial BioF homologues formed a separate, monophyletic group. Given that *N. punctiforme* does not appear to possess any other gene encoding an AOS enzyme, it is altogether unlikely that *N. punctiforme* is capable of synthesizing sphingolipids. In the context of cross-kingdom symbiosis signalling in which sphingolipids are emerging as important regulators, it appears unlikely that sphingolipids from *N. punctiforme* play a regulatory role during its symbiotic association with plants.

## Introduction

**Fig. 1.**
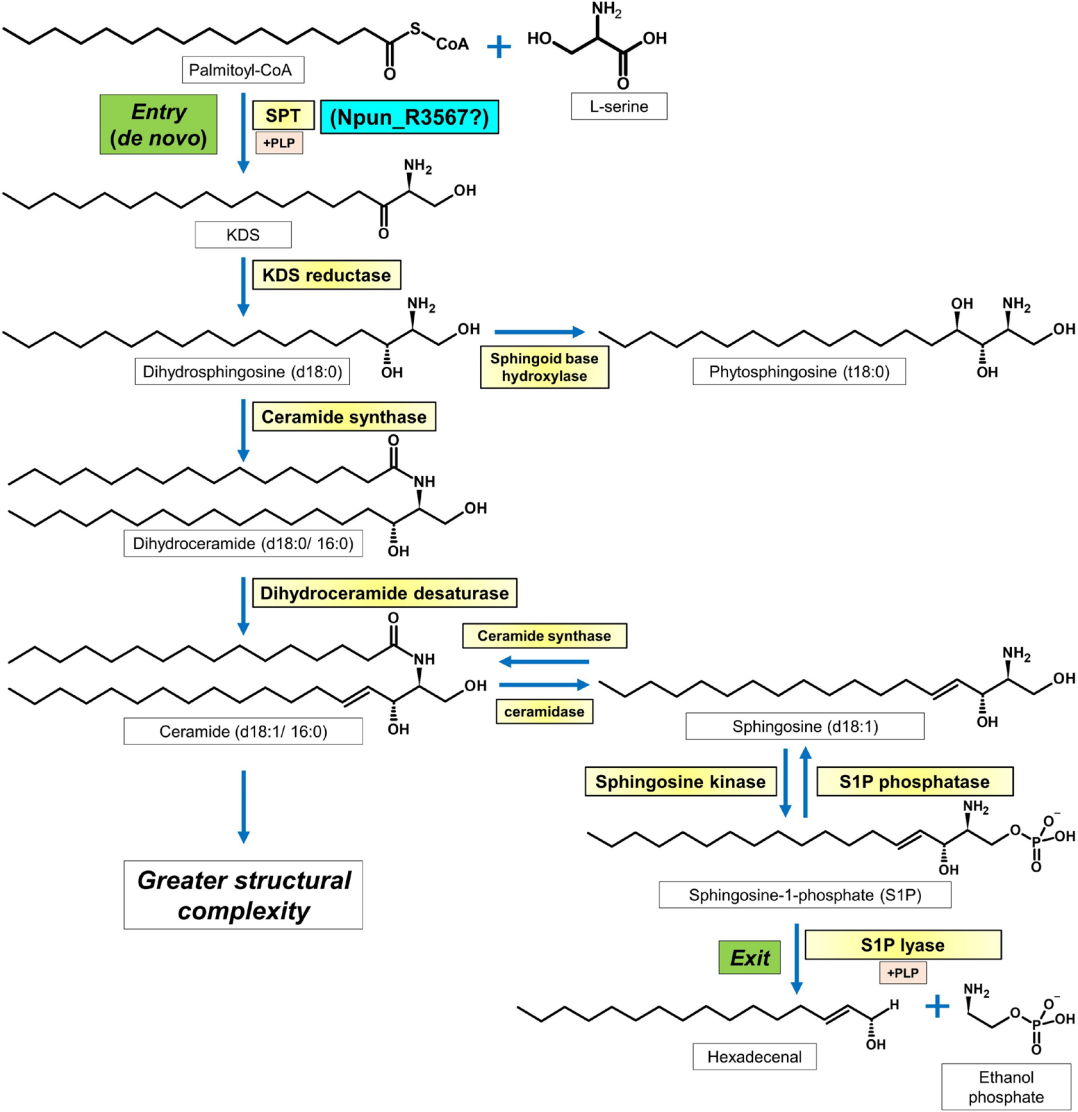
Canonical sphingolipid metabolic pathway. The *de novo* step (entry point) in the pathway, which is catalyzed by serine palmitoyltransferase, is conserved across all sphingolipid-producing organisms. Ceramide (d18 : 1/16 : 0) is a core sphingolipid from which a wide diversity of complex sphingolipids are produced. Alternatively, ceramide may be catabolized into sphingosine (d18 : 1). Phosphorylation of d18 : 1 produces the well-characterized signalling sphingolipid, sphingosine-1-phosphate (S1P). The exit point of the pathway is marked by another pyridoxal phosphate-dependent enzyme, S1P lyase.

Sphingolipids are the third most abundant class of lipids to be found in cell membranes after glycerophospholipids and sterols [1]. Sphingolipids have numerous cellular functions, including lipid ordering [2], signal transduction [3], protein sorting [4] and outer membrane formation (in some gram-negative bacteria) [5]. Additionally, important biological processes mediated by sphingolipids include stress tolerance [6, 7], development [8] and, more recently described, cross-species signalling [9]. In their simplest form, sphingolipids are termed ‘long-chain bases’ (LCBs), which are C14–C20 fatty amino alcohols. Apart from chain length, LCBs can differ by the degree of hydroxylation, desaturation, and the presence or absence of a C1 phosphate and *iso*- or *anteiso*-methyl branches [10]. The most important LCB modification is *N*-acylation with long- or very-long-chain fatty acids to produce ceramides, which are the ‘core’ sphingolipids from which greater structural complexity is built [11] (Fig. 1). C1 head groups, such as sugars and phosphates, can be further added, producing the more soluble ‘complex sphingolipids’ [10, 12, 13].

LCB production begins with the decarboxylative condensation reaction between l-serine and palmitoyl-CoA to produce 3-keto-diyhydrosphingosine (KDS), which rapidly reduces into dihydrosphingosine (d18 : 0). The former reaction is catalyzed by serine palmitoyltransferase (SPT), a member of the small, although widespread family of pyridoxal phosphate (PLP)-dependent α-oxoamine synthase (AOS) enzymes [14–16] (Fig. 1). In eukaryotes, most SPTs are insoluble heterodimers comprising at least two subunits: LCB1 and LCB2 [14, 17]. In contrast, bacterial SPTs are soluble homodimers encoded by the *Spt* gene [18, 19]. This solubility has led to the characterization of several bacterial SPT enzymes [18–20], which are used as models for their eukaryotic counterparts [16].

Despite being ubiquitous in eukaryotes, sphingolipids have only been characterized in a few prokaryotes [12]. The majority of information about sphingolipids in prokaryotes comes from the gram-negative genera *Sphingomonas *, *Sphingobacterium* and *Bacteroides* [12, 21]. Although an acetylsphingomyelin and a cerebroside have been identified in two species of cyanobacteria – *Scytonema julianum* [22] and *Moorea producens* [23], respectively – a sphingolipidome or genetic basis has yet to be reported. To date, no free cyanobacterial LCB (i.e., LCBs not incorporated as part of a ceramide molecule) or sphingolipid enzymes have been characterized.

In eukaryotes, the most common LCBs are dihydrosphingosine (d18 : 0), sphingosine (d18 : 1) and phytosphingosine (t18 : 1), which is a tri-hydroxylated LCB present only in plants and fungi [10, 24] (Fig. 1). Whilst C14, C16, C20 and even odd-chain LCBs have been found [25–27], they appear to be less prevalent. Much less is known about the LCB composition of bacteria. Past research has uncovered both odd- and even-chain LCBs (C16–C19) in prokaryotes, along with unusual branched-chain variants similar to those found in the nematode *Caenorhabditis elegans* [28–31]. The ceramides found in *S. julianum* and *M. producens* are composed of C18 and C17 unbranched LCB backbones, respectively [22, 23].

In this study, efforts were made to characterize sphingolipids in *Nostoc punctiforme* strain PCC 73102 (ATCC 29133), a nitrogen-fixing, plant-symbiotic and cyanobacterial model organism [32–35]. This choice of species was motivated by a rapidly growing body of evidence implicating sphingolipids as important mediators of cross-species signalling in a diverse range of pathogenic and symbiotic relationships [9]. Examples of symbioses in which sphingolipids appear important include those formed between gut *Bacteroides* species and the mammalian gut [6, 36], dinoflagellates and cnidarians [7], Rhizobia and legumes [37], and *Flectobacillus major* (* Bacteroidetes *) and rainbow trout [38]. *Nostoc* species such as *N. punctiforme* can form nitrogen-fixing symbioses with a wide diversity of non-vascular and vascular plant species [34]. *N. punctiforme* strain PCC 73102 was originally isolated from the coralloid roots of a cycad belonging to the genus *Macrozamia* [39], and notably is capable of intracellularly colonizing *Gunnera* species and *Oryza sativa* [40–42]. Moreover, we recently demonstrated that this strain could protect against programmed cell death (PCD) in *Arabidopsis thaliana*, an effect that appeared to be dependent on secreted lipophilic and/or amphiphilic compounds [43]. This suggests that *N. punctiforme* lipids may play a role in regulating PCD, a trait that is hypothesized to be important in symbiosis establishment [7, 44, 45]. If cyanobacteria-derived sphingolipids are important during symbiosis establishment and maintenance, it could be that *N. punctiforme* is capable of sphingolipid production.

Here, we used a sphingolipidomic approach and found that *N. punctiforme* cells do not possess canonical sphingolipids (i.e., unbranched LCBs and ceramides). Keyword and blast sequence analysis of the well-annotated *N. punctiforme* genome [35, 46] using previously characterized bacterial AOS peptide sequences resulted in the identification of only one AOS-family protein (Npun_R3567), which was annotated as an 8-amino-7-oxononanoate synthase (BioF). No phylogenetic evidence supporting an SPT identity for Npun_R3567 could be found, and the protein was unable to produce KDS when heterologously expressed in *Escherichia coli *. This work is the first to attempt a genetic and lipidomic analysis of sphingolipid production in a cyanobacterium. The results provided should inform future avenues of experimental investigation to elucidate sphingolipid production in cyanobacteria.

## Methods

### Strains and growth conditions

*N. punctiforme* strain PCC 73102 (ATCC 29133) cells were maintained under 24 h of continuous cool white fluorescent light (approx. 50 µmol m^−2^ s^−1^) on BG11_0_ medium [39] containing ammonium (4 mM NH_4_Cl, 8 mM TES, pH 7.5) and solidified with 1.5 % BACTO agarose. For experiments, a volume of cells equivalent to 60 µg of chlorophyll *a* was added to 100 ml of fresh growth medium and left to grow for 21 days under a photoperiod of 16 h light (approx. 165 µmol m^−2^ s^−1^) and 8 h dark with constant shaking at 120 r.p.m. The growth conditions for cells used in sphingolipidomic and gene expression analyses were the same, with the exception that gene expression was analysed at different timepoints post-induction of 21-day-old cells (see below). Liquid cell suspension cultures of *A. thaliana* var. Ler-0 were grown under the same conditions but in Murashige and Skoog (MS) medium containing 3 % sucrose, 0.5 mg l^−1^ naphthaleneacetic acid (NAA) and 0.05 mg l^−1^ kinetin (pH adjusted to 5.8 using KOH). Every 7 days, *A. thaliana* cells were sub-cultured by transferring 10 ml of medium with mature cells into 90 ml of fresh MS growth medium.

### Lipid extraction from *N. punctiforme* cells

Four protocols were tested in this work and are outlined in detail in Data Sheet S1 (available in the online version of this article). ‘Method IV’ developed by Markham *et al*. [47] proved most effective at enriching for sphingolipids (Fig. S1, Data Sheet S1). For *N. punctiforme *, lipids were extracted from 500 mg of snap frozen, 21-day-old cells grown in either BG11_0_(NH_4_) or BG11_0_ medium, which is BG11 medium lacking any source of combined nitrogen. As a positive control, lipids were extracted from 120 mg of 7-day-old, lyophilized *A. thaliana* Ler-0 cells. As a negative control, a blank extraction was performed without adding biological material.

All standards were purchased from Avanti Polar Lipids and are listed in Table S2 (Data Sheet S1). As internal standards, d17 : 0, d17 : 1 and d17 : 1-P LCBs were added during each extraction. Where cells were being screened for C17 LCBs, d16 : 1, d18 : 1 and d18 : 1-P were instead added. Standards also allowed for calculation of sphingolipid recovery (*R*), sample matrix effects (*M*) and overall process efficiency (*P*), which are explained in Data Sheet S1. Additional standards used for calibration and standard curve generation included d18 : 0, d18 : 0-P, CerP(d18 : 1/12 : 0-P), GlcCer(d18 : 1/12 : 0), LacCer(d18 : 1/12 : 0) and Cer(d18 : 1/25 : 0). A list of standard retention times, product ions and limit of detection is given in Table S3 (Data Sheet S1).

### LC-ESI-MS/MS analysis of lipid extracts

Chromatographic analyses were performed using an Agilent 1290 Infinity II Ultra-HPLC (UHPLC) system. Lipid extract analysis was carried out on a reversed-phase C8 column (Kinetex 2.6 µm, 100 Å, 100×2.1 mm, Phenomenex; ref. 00A-4497-AN) maintained at 50 °C with a flow rate of 400 µl min^−1^. The mobile phases, consisting of (A) nanopure water with 5 mM ammonium formate and (B) methanol/acetonitrile/isopropanol (10 : 40 : 50, by vol.), 5 mM ammonium formate with 0.1 % formic acid, were pumped into the UHPLC system with the following elution programme: 0–2 min, 40 % B; 2–22 min, 40–90 % B; 22–25 min, 90 % B. Subsequently, the B content was rapidly (1 min) decreased to the initial conditions and the column was re-equilibrated for 5 min before the next acquisition (total run time 31 min). Prior to sample injection, raw extracts were re-dissolved in 500 µl of isopropanol/methanol (2 : 1, v/v) and 500 µl of H_2_O/methanol (2 : 1, v/v).

Mass spectral analyses were performed using an Agilent 6550A iFunnel quadrupole time-of-flight (Q-TOF) mass spectrometer equipped with a Dual Agilent Jet Stream electrospray ionization source (Dual AJS ESI). Mass spectra were acquired in positive ion polarity full scan mode, with a mass-to-charge ratio (*m*/*z*) detection range of 100–1400 selected. The electrospray source parameters were operated as follows: capillary voltage was 3.5 kV with 0 V of collision energy; and gas temperature was 150 °C with a dry gas flow of 14.0 l min^−1^. The nebulizer was set at 40 psi, nozzle voltage was powered at 1.5 kV and fragmentor was 365 V. For internal mass calibration during the MS analysis, reference masses 121.0509 (purine [C_5_H_4_N_4_+H]^+^) and 922.0098 (HP-0921 [C_18_H_18_O_6_N_3_P_3_F_24_+H]^+^) were used.

MS/MS data were generated using a data-dependent strategy, selecting targeted precursor ions listed in Table S4, Data Sheet S1. The mass range detection for collision-induced dissociation (CID) was fixed between 30 and 630 *m*/*z* for LCBs, 50 and 850 *m*/*z* for ceramides, and 100 and 1000 *m*/*z* for neutral glycosphingolipids, in which three microscans at an isolation width of 4 atomic mass units was selected. For each precursor ion, four fixed collision energies were selected (5, 12, 18 and 22 eV).

### Qualitative and quantitative analysis of sphingolipids

UHPLC/Q-TOF-MS/MS data were analysed using the Agilent MassHunter software for qualitative analysis (V. B.07.00). Sphingolipid identification was achieved by comparison with the generated MS-related sphingolipid standard reference information (Table S5, Data Sheet S1) and other MS-related information reported in previous works [48–51]. Putative molecular ions identified within 5 p.p.m. were selected for verification by MS/MS analysis. Sphingolipid concentrations were measured using standard curves generated using sphingolipid standards (Fig. S2, Data Sheet S1). Actual concentrations were estimated after factoring in internal standard recovery levels. However, as cautioned previously [47], absolute concentrations cannot be accurately determined in this way as recovery levels are not equal across all classes of compounds unless standards for each compound analyte are added prior to extraction, which often is not practical. Therefore, concentrations were relative to the mean recovery levels of the three C17 internal standards.

### Differential gene expression analysis

To induce diazotrophy, cells from a 100 ml, 21-day-old (stationary growth; Fig. S7, Data Sheet S2) liquid *N. punctiforme* culture were washed with BG11_0_ medium (-N) to remove ammonium. Then, the culture was split into two 50 ml volumes. To one, fresh ammonium (as above) was added back [BG11_0_(NH_4_)], whereas nothing was added to the other (BG11_0_). Cultures were then transferred to standard liquid culture growth conditions. See Fig. S8 (Data Sheet S2) for an induction workflow.

RNA was isolated using a method developed by Pinto *et al*. [52] and cleaned using an RNeasy plant mini kit (Qiagen). Total RNA (500 ng) was treated with DNase I (Thermo Scientific), and cDNA was synthesized using SuperScript II reverse transcriptase (Invitrogen). For real-time quantitative PCR (RT-qPCR), *Npun_F5020* and *Npun_F5466* (unchanged expression between treatments) were used as housekeeping genes (homologues from *Anabaena* sp. strain PCC 7120 [53], genes *all5167* and *alr0599*, respectively). As a diazotrophy marker, *Npun_F1722* (*hetR*) was used [54]. As a marker of hormogonia, *Npun_F2507* (*pilT*) was used [32]. RT-qPCR was performed using FAST SYBR Green master mix (Applied Biosystems) on a ViiA RT-qPCR instrument (Applied Biosystems). All reactions were performed in triplicate. Relative changes in expression were calculated according to Pfaffl [55]. The primer sequences used in this section are detailed in Table S6 (Data Sheet S2).

### Cloning and heterologous expression of *Npun_R3567* and *SmSpt*

The ORF *Npun_R3567* was PCR-amplified from genomic DNA (gDNA) and cloned into pET21a+, which is part of a bacterial expression plasmid series [pET21a-d(+)] used before to clone bacterial SPTs [18, 56]. This was done as follows: gDNA was isolated according to Tamagnini *et al*. [57]. Gene-length primers were designed to PCR-introduce *Bam*HI (5′) and *Xho*I (3′) restriction sites for insertion upstream of a polyhistidine tag (removing TAA stop codon). The *Spt* gene from *Sphingobacterium multivorum* (*SmSpt*) was restriction-cut from a custom-ordered plasmid (pEX-K4; Eurofins Genomics) harbouring the synthesized gene (*AB259214.1*). Ligation reactions were transformed into *E. coli* strain DH5α before positive selection on ampicillin-containing growth medium (50 μg ml^−1^). The resulting expression plasmids were pUDSB1 (pET21a+w/*Npun_R3567*) and pUDSB2 (pET21a+w/*SmSpt*), which were verified by sequencing (Eurofins Genomics). The primer sequences and plasmids used in this section are listed in Tables S8 and S9 (Data Sheet S3), respectively.

For heterologous expression, plasmids were transformed into the *E. coli* lysogen BLD21(DE3) and induced by adding 0.4 mM IPTG to liquid cultures with an OD_600_ of 0.6 and subsequent incubation for 18 h. A workflow depicting the heterologous expression approach is shown in Fig. S11, Data Sheet S3. Protein synthesis was checked by immunoblotting for the presence of the polyhistidine tag in the soluble protein fraction. To do this, 2 ml of induced cells was centrifuged, flash frozen and resuspended in 600 µl of ice-cold potassium phosphate buffer, as done by Ikushiro *et al*. [56], except that no AEBSF protein inhibitor was used. Cells were lysed by shaking cells for 5 min at 30 Hz in the presence of 300 mg of glass beads (0.1 mm Ø), after which samples were centrifuged at 20 000 ***g*** for 30 min at 4 °C. Immunoblotting was performed by dry-transferring protein from SDS-PAGE gels onto nitrocellulose membranes (Bio-Rad) and incubating in primary [6×-His Epitope Tag mouse monoclonal antibody (ThermoScientific; ref. MA1-21315)] and then secondary [rabbit anti-mouse IgG (H+L) secondary antibody, HRP (ThermoScientific; ref. 61–6520)] antibody. Luminescence of recombinant protein was measured at 428 nm after incubation with enhanced chemiluminescence substrate (Amersham ECL Select).

### Lipid extraction from *E. coli* cells

For *E. coli* cells which were induced for the heterologous expression of *Npun_R3567* and *SmSpt* [18], lipids were extracted using the same protocol as described above for *N. punctiforme* cells. Unlike *N. punctiforme* cells, however, lipids were extracted from snap frozen (rather than lyophilized) cells.

### Sequence analysis of Npun_R3567

For phylogenetic analysis, a suitable set of 28 homologous, non-cyanobacterial, AOS-family peptides were obtained from Geiger *et al*. [58]. These included a number of enzymatically characterized peptides, which were used as blast-search queries to identify 29 putative cyanobacterial AOS-family proteins on GenBank. Finally, an additional four non-cyanobacterial sequences were identified from keyword searches on PubMed, focusing on post-2010 (i.e., post-Geiger *et al*. [58]) enzyme characterization studies. All sequence accession numbers are listed in Table S10 (Data Sheet S4). The sequences varied in length from 313 to 409 residues but features and domains that characterize the sequences as members of the AOS family of enzymes were found in all 60 polypeptides.

A multiple sequence alignment (MSA) of the data was inferred using MAFFT v7.471, using the G-INS-i option, which assumes that all sites can be globally aligned [59, 60]. For functional residue comparisons of Npun_R3567 with enzymatically characterized SPTs, a box-shaded sub-MSA was made using Jalview2 [61]. Before phylogenetic analysis, the MSA was examined. First, AliStat v1.12 [62] was used to assess completeness. This revealed that the MSA was sparce (i.e. containing many alignment gaps and/or ambiguous letters). Because missing data in MSAs may interfere with phylogenetic inference, the sites of the MSA were masked. Using a threshold of C_c_=1.0, all sites which contained alignment gaps and/or ambiguous letters from the original MSA with 527 sites were masked. This produced a sub-MSA with 291 sites. Next, the sub-MSA was surveyed for evidence that the data have evolved under stationary and reversible conditions (most phylogenetic methods assume evolution under such conditions and might return biased results if the data actually had evolved under non-stationary and/or non-reversible conditions [63, 64]). In practice, Homo 2.0 (http://github.com/lsjermiin/Homo.v2.0) [65] was employed to assess whether it was sensible to assume that the sequences evolved under stationary and reversible conditions. This appeared to be the case (i.e., the 5 % family-wise error rate of 0.05/1770 tests was less than the smallest probability of getting a result from the matched-pairs test of symmetry by chance, which was 0.0046).

Having surveyed the data as outlined above, the phylogenetic inference was started. Unlike most phylogenetic studies, the task was not split into a step aimed at finding the optimal model of sequence evolution (SE) for the data, and a step aimed at finding the optimal phylogeny, conditional on the optimal model of SE. Instead, the advanced version of ModelFinder [66] was employed, which allowed both tasks to be performed simultaneously while considering a greater-than-normal set of models of rate-heterogeneity across sites. In practice, the ‘-mrate E,I,G,I+G,R,I+R,H,*H -mfreq FU,F,FO’ options were used. To accommodate both sensitivity and specificity during model selection [67], both the Bayesian information criterion (BIC) [68] and Akaike's information criterion (AIC) [69] optimality criteria were used. Under BIC, the optimal model of SE was LG+FO+I+G4 (138 parameters), and under AIC, it was LG+FO*H5 (684 parameters). Both trees were inferred using IQ-TREE 2 [70] while the consistency of the phylogenetic estimates was inferred using the UFboot method with 10 000 replicates [71]. The most likely trees were visualized using FigTree [72].

## Results

### Identification of an AOS-family homologue in *N. punctiforme*

**Fig. 2.**
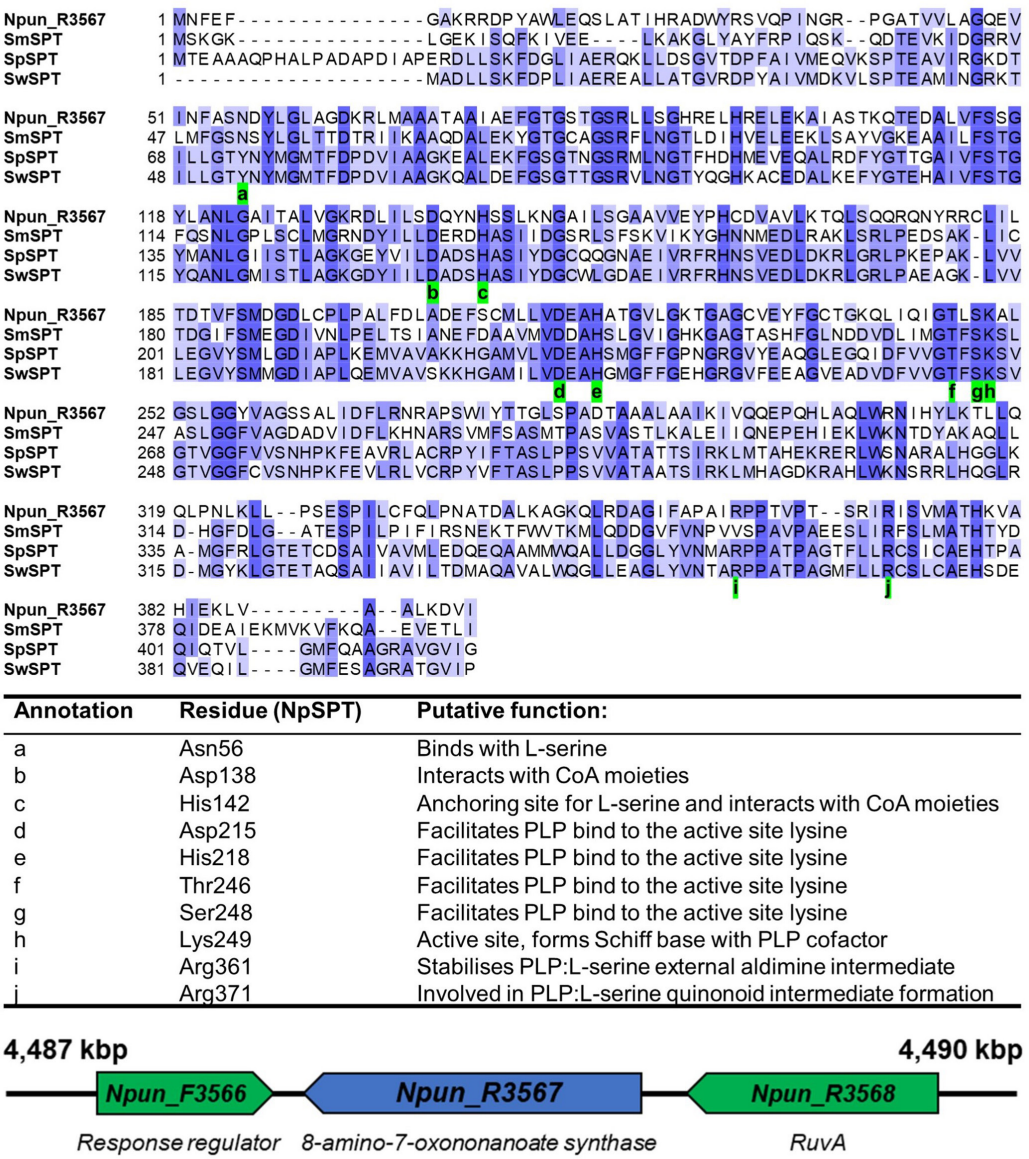
(Top) Alignment of the *N. punctiforme* protein, Npun_R3567, with the peptide sequences of three characterized SPTs from *Sphingobacterium multivorum* (SmSPT), *Sphingomonas paucimobilis* (SpSPT) and *Sphingomonas wittichii* (SwSPT). Indicated are residues (green) in Npun_R3567 which correspond to catalytically important residues in the characterized SPTs (see the table). (Bottom) Genomic context of Npun_R3567, which is annotated as an enzyme which encodes an 8-amino-7-oxononanoate synthase (BioF).

In a step towards identifying a SPT homologue in *N. punctiforme *, eleven enzymatically characterized AOS-family peptides (Table 1), including eight SPTs, were used as blast query sequences to identify an *N. punctiforme* homologous peptide (Npun_R3567) comprising 394 amino acids (Fig. 2). The protein is predicted to function as a BioF, which catalyses the first-step reaction in biotin biosynthesis [73]. However, this is a common annotation for bacterial SPTs [19, 74] and the predicted protein sequence was most similar (38.75 % identity) to the SPT from the gram-negative bacillus *Sphingobacterium multivorum* [18] (Table 1). Alignment of these, along with two other molecularly characterized bacterial SPTs, revealed that Npun_R3567 possesses several conserved residues which, in the other proteins, are associated with the SPT enzymatic mechanism (Fig. 2). Of these, eight are shared by all four proteins, whereas two are shared by two and one other protein, respectively. In the characterized proteins, five of these ten residues are involved in the formation of a Schiff base with the PLP cofactor (internal aldimine), three are involved in the formation of the PLP:l-serine quinonoid intermediate (external aldimine), and two interact with ribose and adenine moieties of acyl-CoA molecules (Fig. 2) [16, 20, 75, 76].

**Table 1.** Results of a blast protein comparison of Npun_R3567 with different enzymatically characterized AOS-family enzymes from bacteria

Species	Enzyme	Length (aa)	*E*-value	Percentage identity
* Sphingobacterium multivorum *	SPT [18]	399	7.6E-92	38.75 %
* Porphyromonas gingivalis* str. W84	SPT [92]	395	1.9E-79	36.12 %
* Bacteriovorax stolpii *	SPT [18]	420	2.4E-79	35.78 %
* Bacteroides thetaiotaomicron *	SPT [74]	394	2.1E-78	37.40 %
* Bacteroides fragilis* str. S23L17	SPT [6]	394	1.8E-76	33.88 %
* Escherichia coli *	KBL [93]	398	2.9E-67	33.08 %
* Caulobacter crescentus *	SPT [94]	404	5.8E-61	32.38 %
* Sphingomonas wittichii *	SPT [19]	400	1.1E-58	30.65 %
* Sphingomonas paucimobilis *	SPT [56]	420	3.3E-57	31.05 %
* Rhodobacter capsulatus *	HemA [95]	401	2.1E-54	33.16 %
* Escherichia coli *	BioF [73]	384	2.4E-48	32.97 %

### Sphingolipidomic profiling of *N. punctiforme* cells

**Fig. 3.**
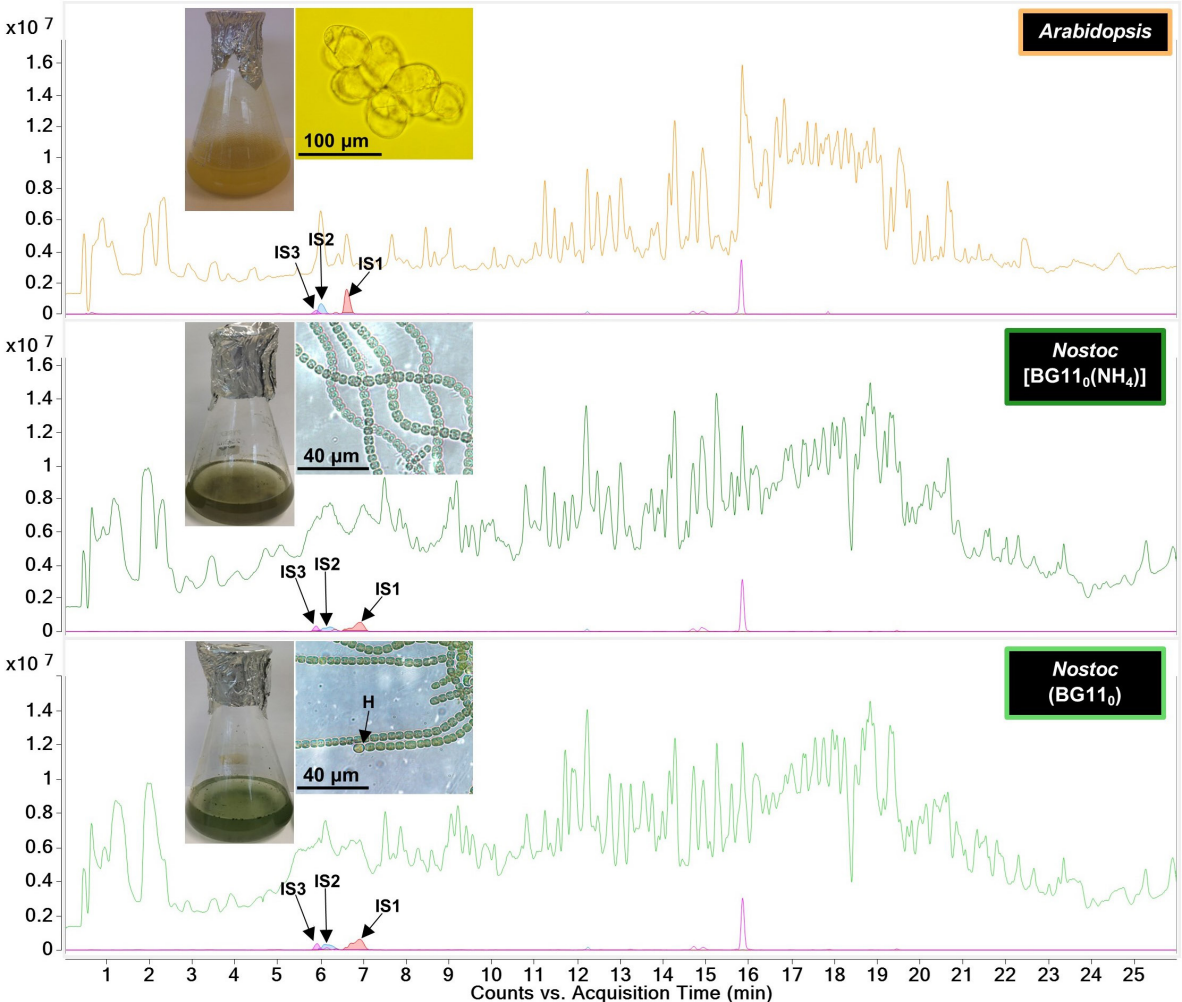
Total ion chromatograms generated by UHPLC/Q-TOF-MS analysis of lipid extracts from *A. thaliana* and *N. punctiforme* cells. The latter were cultured with and without a source of combined nitrogen. Diazotrophic growth is marked by the formation of heterocyst (H) cells. Indicated are the extracted ion chromatograms for the three LCB internal standards (IS), which are d17 : 0 (IS1), d17 : 1 (IS2) and d17 : 1 P (IS3). *n*=4.

As no sphingolipid extraction protocol was available for cyanobacterial cells, we tested four lipid extraction protocols and selected one which is based on a method developed by Markham *et al*. [47] to enrich for both polar and non-polar sphingolipids from plant cells (Fig. S1, Data Sheet S1). This was due to its superior levels of internal LCB standard recovery, which ranged between 10 and 20 % (Table S1, Fig. S3, Data Sheet S1). Using a UHPLC/Q-TOF-MS/MS method, internal standards eluted between 4 and 8 min on a C8 reversed-phase column, as shown for the C17 internal standards in Fig. 3. An overall extraction process efficiency of 10.3±1.6% and an instrument loss on detection of ~1 ng ml^−1^ for commercial C16, C17 and C18 LCBs (Table S3, Data Sheet S1) indicated that we would only have failed to detect LCBs if they naturally occurred at a concentration of less than 9.7 ng ml^−1^, equivalent to an ~32 fM concentration of d18 : 0. As a positive control for natural sphingolipid recovery, undifferentiated *A. thaliana* Ler-0 cells grown in liquid suspension were screened. In these cells, t18 : 0, which was the most abundant LCB detected, was estimated to occur at an ~3 pM concentration (Figs S4 and S6, Data Sheet S1). Indeed, the method allowed for the successful identification of several C18 LCBs and ceramides in *A. thaliana* cells (Table S5, Fig. S5, Data Sheet S1).

To screen for sphingolipids in *N. punctiforme* cells, lipids were extracted from both vegetative and diazotrophic (involving heterocyst differentiation) cultures that were 21 days old, which was approximately the beginning of the stationary growth phase for cells grown with and without a source of combined nitrogen (Fig. S7, Data Sheet S2). However, no sphingolipids could be detected under either growth condition. To control for the possibility that *N. punctiforme* LCBs could be C17-based, extractions were then repeated using C16 and C18 as internal standards, but none were detected. Finally, using the METLIN database integrated into the Agilent MassHunter data analysis software, we used an algorithm to search for more atypical sphingolipid classes. Considering all possible charge carriers (i.e. sodiation and protonation), dimers, in-source fragmentation losses (H_2_O and H_3_PO_4_) and charge ranges yielded no sphingolipid matches.

### Gene expression analysis of *Npun_R3567*

**Fig. 4.**
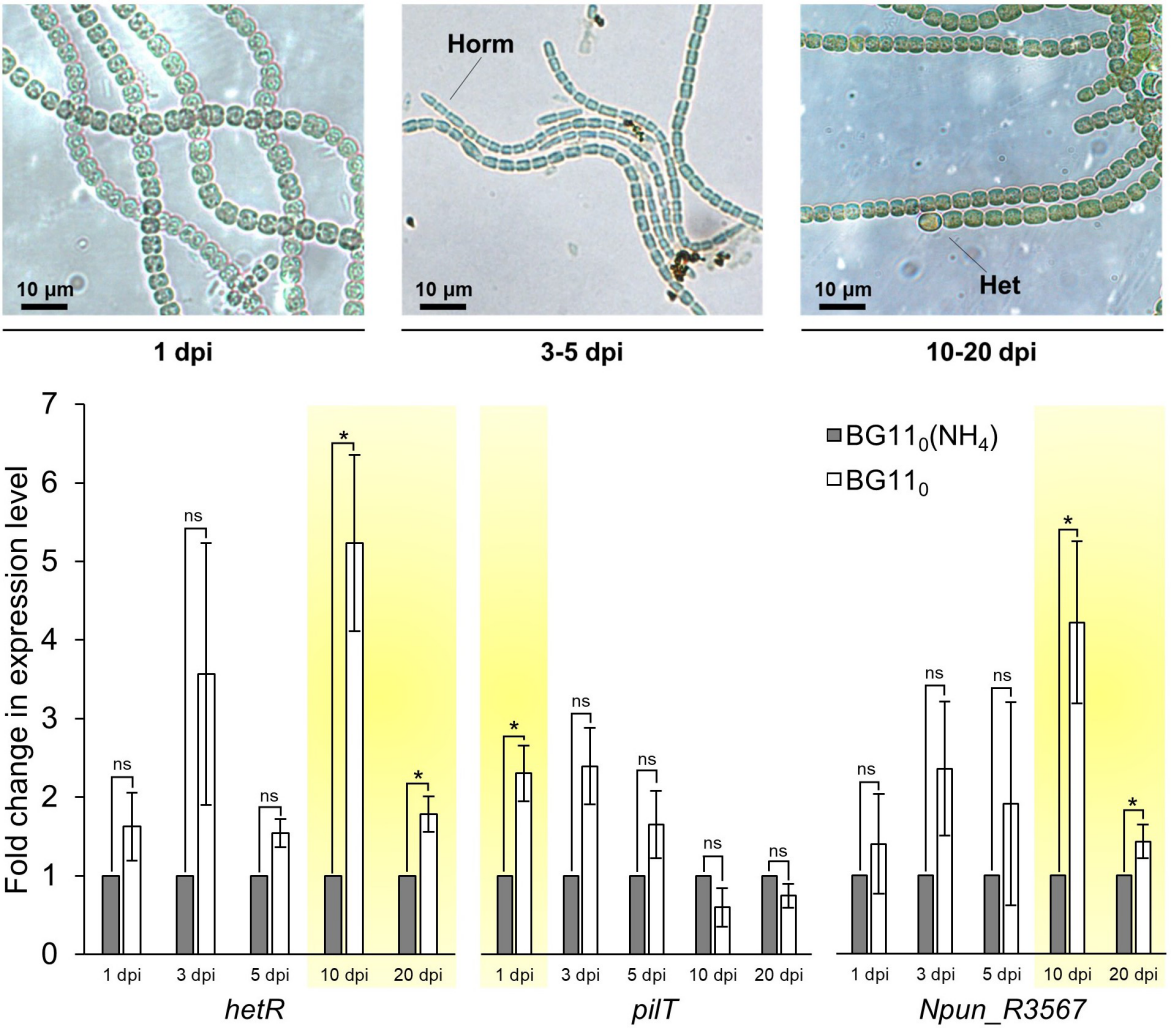
(Top) Representative *N. punctiforme* cells imaged at various days post-induction (dpi) for diazotrophy. At 1 dpi, only vegetative filaments are observable. Between 3 and 5 dpi, hormogonia cells (Horm) are observable. Between 10 and 20 dpi, mature heterocyst (Het) cells are differentiated. (Bottom) Relative change in expression levels of *hetR* (marker for heterocyst formation), *pilT* (marker for hormogonia formation) and *Npun_R3567* at different days post-induction (dpi). Significant differences (yellow shading) between treatments were determined using a Student’s *t*-test (*P≤0.05). Values are means±se, *n*=5.

The expression of *Npun_R3567* was measured at 1, 3, 5, 10 and 20 days post-induction (dpi) for diazotrophy (Fig. 4). In response to nitrogen stepdown, hormogonia cells transiently differentiated (3–5 dpi), followed by heterocyst formation, which were observed at 10 and 20 dpi (Fig. 4, Table S7, Data Sheet S2). These responses were mirrored in an initial, transient up-regulation of *pilT* at 1 dpi, and a subsequent up-regulation of *hetR* at 10 and 20 dpi. No significant changes in the expression of *Npun_R3567* were observed between 1 and 5 dpi, but at 10 and 20 dpi the gene was significantly up-regulated. The diazotrophy-induced changes in expression of *Npun_R3567* therefore mirrored that of *hetR* (Fig. 4). These results indicate that Npun_R3567 may be important under conditions of diazotrophic growth.

### Npun_R3567 does not produce KDS when heterologously expressed in *E. coli*

**Fig. 5.**
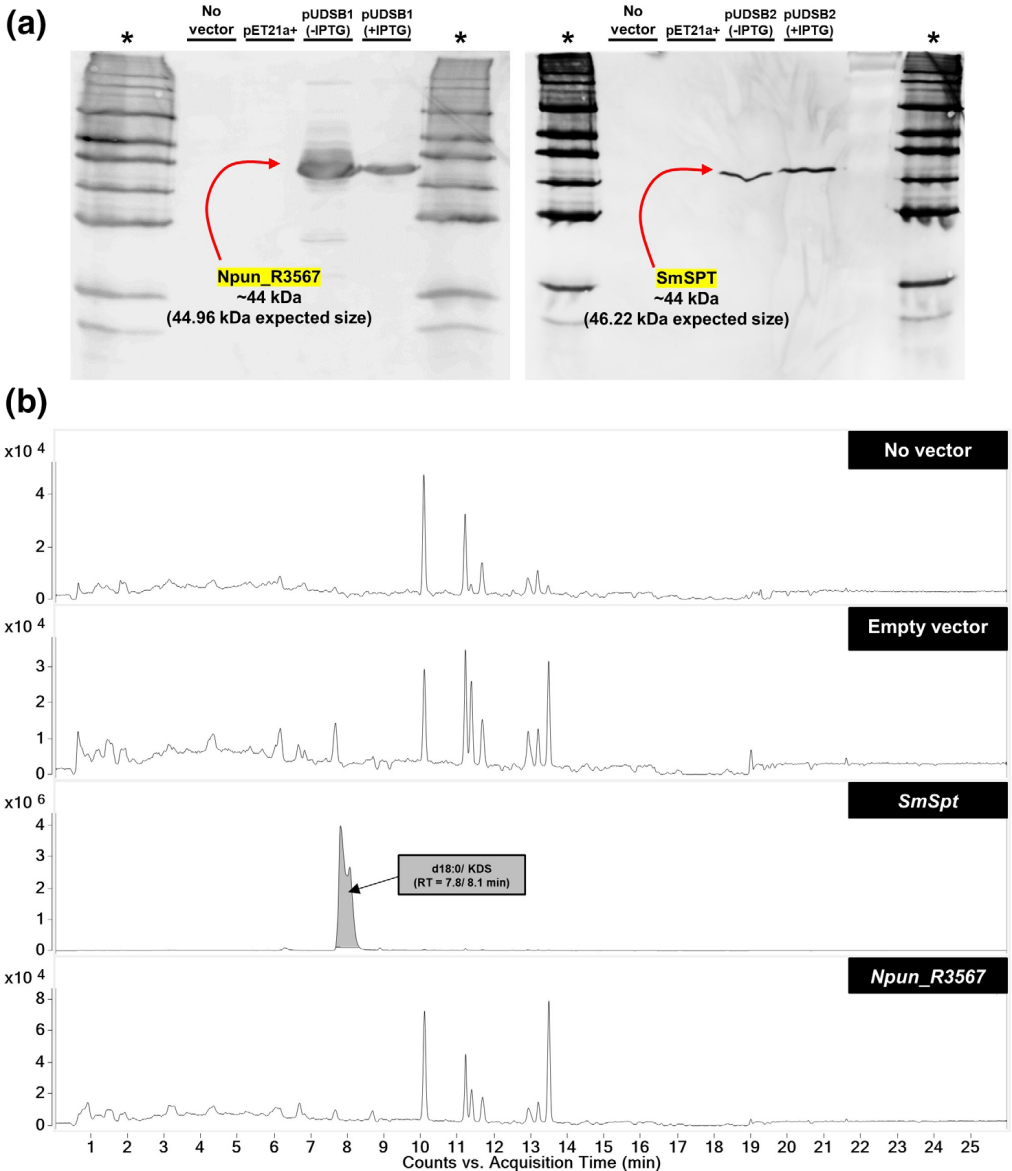
(**a**) Immunoblot detection of recombinant Npun_R3567 (left) and SmSPT (right) in the soluble protein fraction of induced *E. coli* BL21(DE3) harbouring pUDSB1 and pUDSB2, respectively. Negative controls include protein from cells harbouring no vector and an empty pET21a+vector, respectively. *A BenchMark His-tagged protein standard ladder (ThermoScientific) was included to estimate protein size. (**b**) Extracted ion chromatogram generated by UHPLC/Q-TOF-MS analysis of lipid extracts from *E. coli* cells induced for heterologous expression of SmSpt and Npun_R3567. No vector and empty vector controls are also shown. A split peak identified at a retention time (RT) of 7.8 and 8.1 min correspond to d18 : 0 and KDS, respectively. *n*=3.

Although *Npun_R3567* would have been expressed at the timepoint of lipid extraction in our sphingolipidomic screen, silencing at the post-transcriptional level could not be ruled out. To get around this, we used the UHPLC/Q-TOF-MS workflow to test if the SPT reaction product, KDS, would be produced in *E. coli* cells heterologously expressing the Npun_R3567 protein, as most *E. coli* strains do not possess an *Spt* gene [58]. *SmSpt* was expressed as a positive control and both genes were cloned such that they could be expressed as recombinant proteins bearing respective C-terminal polyhistidine tags. This allowed for their immunoblot detection in the soluble protein fraction of induced cells. Chemiluminescence was similar for both proteins in the presence of IPTG (Fig. 5a). However, signals were also detected in the absence of IPTG, indicating promoter leakiness. In this case, a stronger signal was detected in cells harbouring *Npun_R3567*. This may be explained by the formation of insoluble inclusion bodies under inducing conditions leading to a weaker signal. This was supported by immunoblotting the total crude cellular fraction, in which case a significantly stronger signal was detected in the induced cells (Fig. S12, Data Sheet S3).

**Fig. 6.**
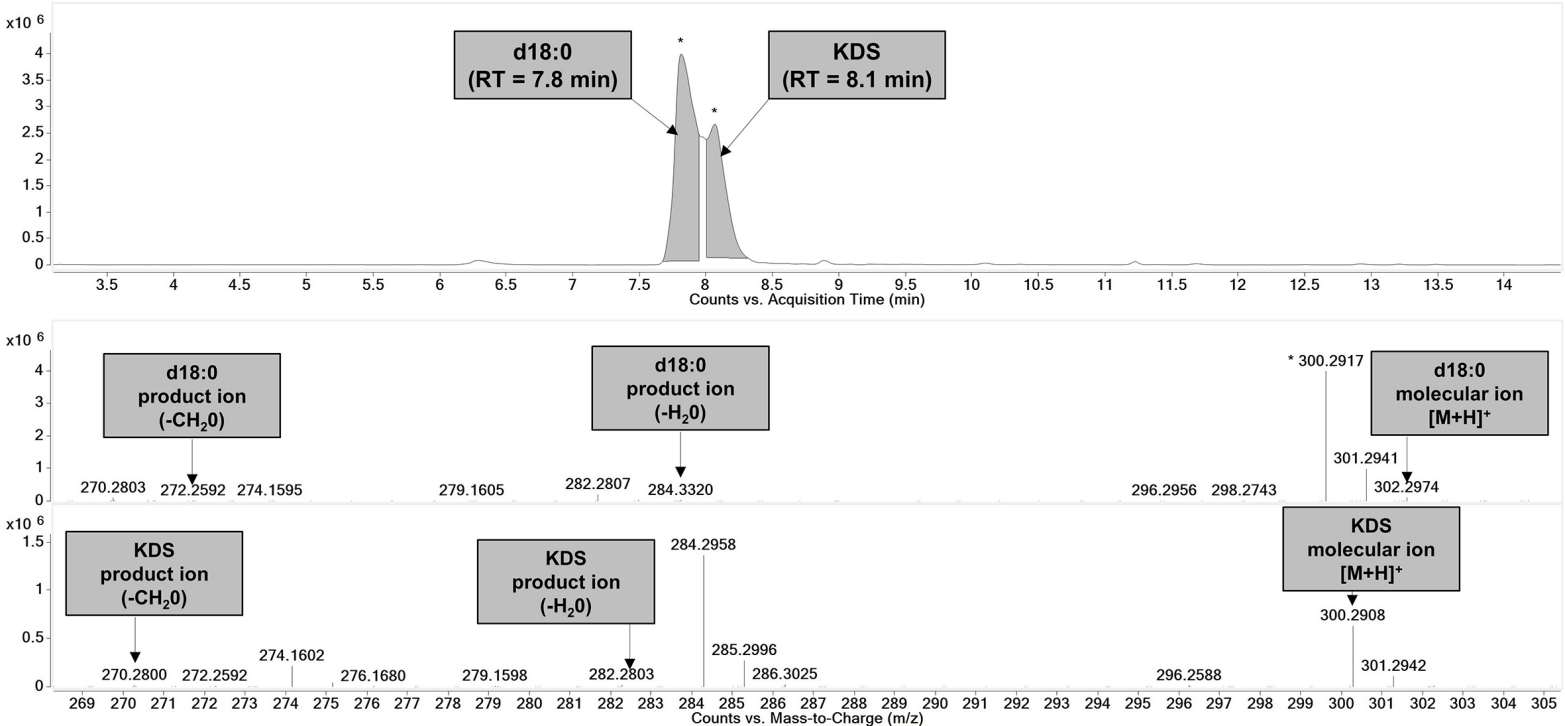
Identification of KDS in lipids extracted from *E. coli* heterologously expressing the previously characterized bacterial SPT from *Sphingobacterium multivorum* (SmSpt). (Top) EIC split peak corresponding to two coeluted compounds, d18 : 0 and the SPT reaction product, KDS (*n*=3). (Bottom) Extracted mass spectra from each peak show the presence of the diagnostic product and molecular ions derived from in-source fragmentation during full scan analysis.

Nonetheless, the level of solubilization achieved under inducing conditions allowed for the detection of a large, split chromatographic peak in the soluble protein fraction of cells expressing *SmSpt* (Fig. 5b). The first shoulder peaked at ~7.8 min, which closely matches the retention time of the commercial d18 : 0 standard, suggesting that KDS may have been reduced by endogenous *E. coli* reductases. Inspection of the mass spectra for this peak revealed product ions from neutral water and formaldehyde loss, as well as a molecular ion ([M+H]^+^) corresponding to d18 : 0 (28 p.p.m. mass error). We infer the second shoulder, which peaked at ~8.1 min, to represent KDS. Although we did not have access to a KDS commercial standard, sphingosine (d18 : 1), which is isomeric with KDS, eluted at 7.16 min. However, d18 : 1 is C4-desaturated, which probably reduces affinity with the C8 column compared to the fully saturated KDS. Therefore, we can expect KDS to elute later than the d18 : 1 standard. Observed product and molecular (3.7 p.p.m. mass error) ions in the mass spectra of the extracted ion chromatogram (EIC) peak at 8.1 min confirmed that KDS was indeed produced by SmSPT (Fig. 6). We estimated that the KDS/d18 : 0 mix was produced at an ~2 nM level (19.6 µmol g dry weight^−1^). By contrast, neither KDS nor d18 : 0 could be detected in cells expressing *Npun_R3567*, which suggests that Npun_R3567 is not an SPT.

### Phylogenetic position of Npun_R3567

**Fig. 7.**
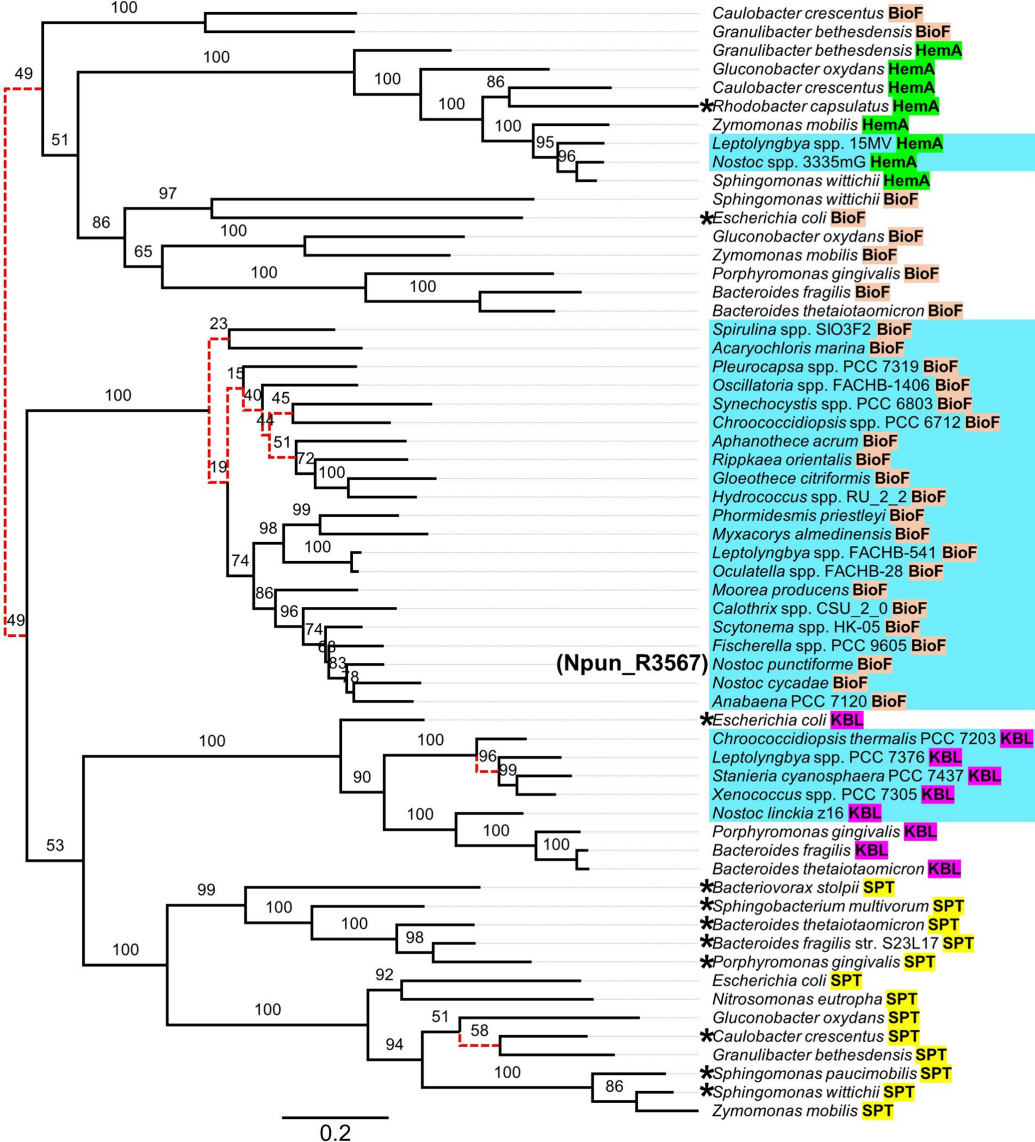
Unrooted phylogeny of prokaryotic AOS-family enzymes inferred using maximum-likelihood under the LG+FO*H5 model of SE (optimal under AIC). Estimates of consistency (%) for internal edges were inferred using the ultrafast bootstrap method (*n*=10000). Using the LG+FO+I+G4 model of SE (optimal under the AIC), the phylogeny differed from the tree shown here at the 10 red, dashed edges. Cyanobacterial sequences are coloured in cyan. Sequences which have been enzymatically characterized are marked with asterisks. Bar, 0.2 substitutions per site. Branch lengths are weighted average over the different heterotachy classes (Table S11, Data Sheet S4).

To determine the phylogenetic position of Npun_R3567, we performed a maximum-likelihood-based phylogenetic analysis of the SPT and non-SPT AOS-family enzymes (Fig. 7). Together, this included 60 sequences predicted to function as either SPT, BioF, 2-amino-3-ketobutyrate coenzyme A ligase (KBL) or 5-aminolevulinate synthase (HemA) enzymes, which are the best-characterized members of the AOS enzyme family [12]. Optimal phylogenetic trees were inferred under two models of SE (i.e., identified using the AIC and BIC optimality criteria in order to accommodate sensitivity and specificity during model selection). Both models identified the LG amino acid substitution model [77] and optimized state frequency by maximum likelihood (FO). AIC identified a heterotachous model of rate-heterogeneity across sites [78] with five classes of sites (H5) (Table S11, Fig. S13, Data Sheet S4), whereas BIC assumed a proportion of invariable sites (I=4.96 %) and a discrete Γ distribution of variable sites with four equally probable classes (G4) and a shape parameter of 1.2128. The most likely trees inferred under these models of SE differed at 10 internal edges, being present in one tree and absent in the other (Fig. 7). Both models revealed three main peptide groups, which can be seen in the tree shown in Fig. 7 (estimated under the LG+FO*H5 model of SE). The first group consisted of the non-cyanobacterial BioF peptides and the HemA peptides. This group was related to the second and third groups. Although these latter two were inferred to be a monophyletic group, this was not well supported (i.e., false positive rate for the group is more than 5 % [71]). The second group was a well-supported monophyletic group of cyanobacterial BioF peptides, whereas the third group comprised two well-supported groups of KBL and SPT peptides, respectively. These groups were estimated to be monophyletic, but this was also not well supported.

## Discussion

### *N. punctiforme* does not produce unbranched sphingolipids

To the best of our knowledge, the biosynthesis of sphingolipids, which are the third most common class of lipids in eukaryotic cells, has not yet been studied in a cyanobacterium. In this work, we investigated whether *N. punctiforme* strain PCC 73012 (ATCC 29133), a nitrogen-fixing, plant-symbiotic and cyanobacterial model organism, produces sphingolipids. A preliminary blast analysis suggested that *N. punctiforme* may possess the first-step enzyme in the *de novo* sphingolipid biosynthesis pathway, SPT (Fig. 2). This served as the basis to develop and implement a sphingolipidomic screening method to search for sphingolipids in *N. punctiforme* cells. The method, which consisted of a sphingolipid enrichment protocol developed by Markham *et al*. [47] coupled with a UHPLC/Q-TOF-MS/MS experimental design, enabled the successful identification of LCBs and ceramides in *A. thaliana* but not *N. punctiforme* cells. This is despite the fact that *Npun_R3567*, which we had hypothesized to encode an SPT, was expressed at the time of lipid extraction (Fig. 4). Moreover, it was also up-regulated in diazotrophic cultures, which agrees with earlier transcriptomic studies in which the gene was shown to be regulated by combined nitrogen depletion [79–82].

It is worth mentioning that our sphingolipidomic analysis focused on the more widespread, unbranched sphingolipids which are based on C14–C20 LCBs. This was based on *a priori* knowledge that unbranched C17 and C18 LCBs form the backbone of ceramides identified in the cyanobacteria, *M. producens* and *S. julianum*, respectively [22, 23]. Methyl-branched LCBs were not manually mined for in the LC/MS data. However, although they appear to be much less common than unbranched LCBs, branched sphingolipids can still be found across kingdoms. For example, C17 methyl-branched LCBs are produced by *Sphingobacterium* species [31] and *Caenorhabditis elegans* [83, 84], whereas a C19 methyl-branched LCB is produced by a marine strain of the fungus *Aspergillus niger* [85]. Iso- and anteiso-branched fatty acids do occur in *Nostoc* and *Anabaena* species [86]. If *N. punctiforme* cells naturally produce branched LCBs, which are often isomeric with unbranched LCBs, then our sphingolipidomic screen would not have detected them because they would have displayed different retention times on the C8 column.

Application of our sphingolipidomic method to *E. coli* cells heterologously expressing the previously characterised *SmSpt* [18] revealed that KDS was produced at a high level (Fig. 7). In contrast, the absence of KDS in *E. coli* cells expressing *Npun_R3567* suggests that Npun_R3567 is not an SPT. This agrees with our sphingolipidomic analysis of *N. punctiforme* cells, which revealed that no sphingolipids were present. However, whilst Npun_R3567 is probably not an SPT, it is worth considering two possible reasons which may have precluded it from producing an LCB precursor molecule in *E. coli *. First, unique *N. punctiforme* interacting proteins not present in *E. coli* may be required for any SPT activity by Npun_R3567. Raman *et al*. [19] observed that the SPT from *Sphingomonas wittichii* (SwSPT) co-expressed with an acyl carrier protein (ACP), which they hypothesized may function as an alternative acyl thioester to CoASH. Whilst such proteins may be required by Npun_R3567, the *Npun_R3567* gene does not appear to be part of an operon containing any gene coding for an ACP. Moreover, of all the bacterial SPT homodimers so far characterized, all can still produce a KDS product in the absence of any co-expressed proteins. This suggests that even in the absence of interacting proteins, if Npun_R3567 were a functional SPT, a KDS product should still have been detectable. Second, because we cannot rule out that *N. punctiforme* cells contain the rarer, methyl-branched LCBs, we also cannot rule out that Npun_R3567 can only produce an LCB precursor molecule using branched acyl-CoAs. *E. coli* does not produce branched-chain fatty acids [87], indicating that supplementation of cells with branched acyl-CoAs may have been necessary to observe a product. Therefore, we can only report that *N. punctiforme* does not produce the more common, unbranched sphingolipids and that future works focusing on characterizing sphingolipids in *N. punctiforme* and closely related taxa should instead prioritize screening for branched-chain LCBs and ceramides.

### What is the enzymatic identity of Npun_R3567?

The first-step enzyme in the *de novo* sphingolipid pathway, SPT, has been well-characterized in several species belonging to the phyla *Bacteroidetes* and *Proteobacteria* [18, 19, 56, 74]. The SPT peptide sequences from these species in addition to other bacterial AOS-family peptides allowed us to conduct a homology-based search of GenBank. This led to the identification of what we hypothesized to be an *N. punctiforme* SPT (Npun_R3567) based on its possession of amino acid residues which in the query sequences are critical for the SPT reaction mechanism (Fig. 2). For example, His159 in the SPT from *Sphingomonas paucimobilis* (SpSPT) was shown previously to be the anchoring site for the PLP–l-serine external aldimine and a residue which interacts with the carbonyl groups of palmitoyl-CoA and KDS [88]. This residue is probably functionally homologous with His142 in Npun_R3567. Also present is a residue (Lys249) corresponding to Lys265, which is the active site in SpSPT. However, other AOS-family enzymes (BioF, KBL and HemA) shared a similar level of homology with Npun_R3567 (Table 1), which was the only identifiable AOS protein in the *N. punctiforme* genome.

The annotation of Npun_R3567 as a BioF coupled with the observation that BioF homologues could also be blast-identified in other cyanobacteria prompted us to include them in a phylogenetic analysis of bacterial AOS-family enzymes. Phylogenies inferred under the optimal AIC- and BIC-predicted models of SE indicate that the cyanobacterial BioF homologues are a well-conserved monophyletic group, even more so than the non-cyanobacterial BioF peptides. The reliability of the phylogenetic estimate is evident in the well-supported HemA, KBL and SPT subgroups, which clustered based on enzyme identity (predicted or characterized) and not based on taxonomic relatedness. By contrast, the non-cyanobacterial BioF peptides were not as closely related, and under BIC, two sequences from *Caulobacter crescentus* and *Granulibacter bethesdensis *, respectively, were instead positioned as part of a monophyletic group with the cyanobacterial sequences. This greater variation in the BioF sequences may be explained by a greater freedom of the BioF peptides to evolve within their respective taxonomic lineages without losing BioF functionality. In this case, stronger taxon-specific signals may impose a masking effect on the functionally important residues. This, in turn, could explain why the cyanobacterial BioF peptides do not cluster with the non-cyanobacterial sequences.

The extent to which this phylogeny is correct depends on how likely it is that we have accurately accounted for the evolution of these sequences. The evolutionary processes inferred to have acted on the sites in the sequences differ in an important and informative manner. The selected model of SE by BIC was LG+FO+I+G4, which was significantly better than the LG+FO model ( Δ BIC=1927.83), indicating that rate-heterogeneity across sites is likely to be a factor that has played a significant role in the evolution of these sequences, as would be expected from a biochemical perspective. However, the LG+FO+I+G4 model assumes homogeneity of site-specific rates of change across lineages, which is unrealistic from a biochemical perspective. To accommodate this, we used the AIC, which is a more sensitive optimality criterion. According to the estimates obtained using AIC, the best model of rate-heterogeneity across sites is heterotachous with five classes of sites, implying a combination of rate-heterogeneity across sites and across lineages. Furthermore, parameters for the five classes of sites are unlinked, implying a large increase in the number of parameters to optimize (including marginal frequencies and edge lengths). Shown in Table S11 (Data Sheet S4) are the marginal frequencies for the five site classes inferred under the LG+FO*H5 model along with the corresponding frequencies inferred under the LG+FO+I+G4 model. The table reveals large differences across the classes of sites in the relative frequencies of some amino acids (e.g. Gly) and small differences for other amino acids (e.g. His). This is consistent with the notion that the amino acids are distributed non-uniformly across the sites and that the majority (possibly all) of the peptides serve a functional purpose. Fig. S13 (Data Sheet S4) shows the edge lengths for the five site classes inferred under the LG+FO*H5 model. By comparing the lengths of identical edges from the five subfigures, it is clear the rate of evolution over a given edge varies considerably for many of the edges, so rate-heterogeneity across lineages is likely to be a factor that has played a major role in the evolution of these sequences. This is a far more realistic evolutionary scenario, even from a biochemical perspective.

In support of the large cyanobacterial group being a conserved cluster of BioF enzymes is the recent characterization by Sakaki *et al*. [89] of the second-step enzyme in the *de novo* biotin biosynthesis pathway in the model cyanobacterium, *Synechocystis* sp. strain PCC 6803. This finding gave good reason for the authors to identify the *Synechocystis* homologue for Npun_R3567 as a *bona fide* BioF, which is the first-step enzyme in the *de novo* pathway [15]. Therefore, based on sequence homology, there is no reason to suspect that the BioF annotation of Npun_R3567 is incorrect. Given that we could find no other AOS-family enzyme in the *N. punctiforme* genome, it suggests that there is no genetic basis for *de novo* sphingolipid biosynthesis in *N. punctiforme *. Finally, it is worth mentioning that unlike *N. punctiforme* strain PCC 73102, the two cyanobacteria in which ceramide-type sphingolipids have been identified – *M. producens* and *S. julianum* – appear to possess more than one AOS sequence. For example, on CyanoBase [90], blast analysis of *M. producens* using SmSPT retrieves a second BioF sequence (LYNGBM3L_27370) of 571 aa in length. It should be of interest in the future to test whether this and other AOS homologues in cyanobacteria can produce KDS when heterologously expressed in *E. coli *.

## Conclusions

This work provides evidence which strongly suggests that sphingolipid production does not occur in the model plant-symbiotic cyanobacterium, *N. punctiforme* strain PCC 73102 (ATCC 29133). Our data show that unlike the cyanobacteria *S. julianum* and *M. producens *, *N. punctiforme* does not possess the more common, unbranched sphingolipids. In addition, sphingolipid production in this species seems altogether unlikely given that the only AOS-family protein annotated in the *N. punctiforme* genome, Npun_R3567, is phylogenetically distinct from bacterial SPTs and cannot produce the SPT reaction product, KDS, when expressed in *E. coli *. Therefore, despite the emerging body of research which is implicating sphingolipids as key signalling molecules in cross-species communication [7, 9, 91], this work indicates that sphingolipid signalling in *N. punctiforme* is not an aspect of the symbiotic associations which this species forms with multiple plant taxa. However, we also must point out that our approach would not have been able to identify the less common, methyl-branched LCBs and their ceramide derivatives. Therefore, although it seems unlikely that *N. punctiforme* produces sphingolipids, we suggest that future workers instead focus their efforts on these structurally rare sphingolipids in *Nostoc* species. Characterizing the AOS homologues in *S. julianum* and *M. producens* should also be of interest, as these are the only cyanobacteria in which sphingolipids have so far been identified.

## Supplementary Data

Supplementary material 1
