# Supplementary material for: Genetic and lipidomic analyses suggest that Nostoc punctiforme, a plant-symbiotic cyanobacterium, does not produce sphingolipids
Source: Access Microbiol. 2022 Jan 21;4(1):000306. doi: 10.1099/acmi.0.000306 (PMC8895605; doi:10.1099/acmi.0.000306)
Supplement: Supplementary material 1 [file acmi-4-0306-s001.pdf]

Contents

Supplementary Data Sheet 1 2
Supplementary Data Sheet 2 19
Supplementary Data Sheet 3 26
Supplementary Data Sheet 4 31

Supplementary Data Sheet 1

Four different lipid extraction methods used:

1) "Bligh and Dyer" method [1]

This is a total lipid extraction method. To begin with, 30 mg of lyophilised *N. punctiforme* cells were dissolved in 2 ml of 100% UHPLC-grade methanol containing 10 mM ammonium formate (pH = 7). A blank extraction and 30 mg of lyophilised *A. thaliana* Ler-0 cells were included negative and positive controls, respectively. At this point, 500 ng of d17:0 and d17:1, and 2,000 ng of d17:1-P were added as internal standards. Once added, vials were vortexed vigorously for 1 minute, sonicated at 45 kHz for 30 min 4°C and extracted overnight in the dark at room temperature (gentle agitation at 75 rpm). The next day, vortex and sonication steps were repeated before centrifuging at 1,200 *g* for 15 min. From this, the supernatant was transferred into a clean vial and the pelleted debris was re-extracted but this time only for 1 hr. After centrifuging, the supernatant was combined with the previous one. For a third extraction, the pelleted debris was dissolved in 2 ml of $CHCl_3$/ methanol (2:1 v/ v; prepared fresh) and vortexed for 1 minute. For phase separation, 1.2 ml of ultrapure H_2O was added before centrifuging as before. From this, the lower phase was carefully removed and combined with the previous two methanol parts. After combining, the sample (~5.3 ml) was dried under a nitrogen stream (~2 hr until completely dry) and stored at -20°C overnight (or worked with immediately). The next day, the extract was dissolved in 2 ml of $CHCl_3$ and 1 ml of 1 M KCl (1st wash step) before vortexing for 1 minute and sonicating for 3 min at 4 KH/z. To phase separate, vials were then centrifuged at 4,000 rpm for 5 min. From this, the upper KCl aqueous phase was removed and discarded. Next, 2 ml of ultrapure H_2O was added (2nd wash step). After vortexing again for 1 minute, vials were once more centrifuged at 4,000 rpm for 5 min. From this, the lower $CHCl_3$ layer was carefully transferred into a new vial and dried under a nitrogen stream. The final extract was then stored at -20°C until UHPLC-Q-TOF-MS analysis.

2) "Folch" method [2]

This is a total lipid extraction method. The protocol used in this work is as follows. Firstly, instead of working with snap-frozen or lyophilised cells, 4 × 100 ml, 21-day-old liquid cultures were harvested by centrifugation at 4,000 rpm for 5 min at room temperature before washing twice with 0.1 M PBS (pH= 7.4). As before, a negative

(no biomass) and positive (30 mg dried *A. thaliana* Ler-0) control were included. After thoroughly removing all PBS from the *N. punctiforme* cell pellet, cells were weighed and the samples resuspended in 2 ml 100% UHPLC-grade methanol and transferred to 7 ml glass vial using glass pipettes. Next, internal standards were added (500 ng of d17:0/d17:1 and 2,000 ng of d17:1-P), after which samples were vortexed vigorously for 30 sec. To homogenise, samples were sonicated in an ice bath for 10 min at 40 kHz. To pellet debris, samples were centrifuged at 4,000 rpm for 5 min at room temperature. From this, the organic phase was transferred to a new glass vial. The pellet was then resuspended in 3 ml of $CHCl_3$/ methanol (2:1 v/ v). Once resuspended, samples were vortexed and sonicated, as before. After this second homogenisation, debris was pelleted again by centrifugation and the organic phase supernatant combined with the previous one. To this organic phase mixture, a ¼ volume of 0.88% KCl was added. Once added, samples were vortexed for 30 sec and centrifuged, as before. From this, the upper aqueous phase was discarded, leaving behind the organic phase to which ¼ volume of methanol/ water (1:1 v/ v) was added. As before, samples were then vortexed and centrifuged, discarding the upper aqueous phase once again. The remaining organic phase was then transferred to a new vial and dried under a nitrogen stream (~2 hr). Once dried, the sample was stored at -20°C for later analysis.

3) "Merrill" method [3, 4]

This is a lipid extraction method designed to enrich for sphingolipids. This method was used in this work as follows. 100 ml of 21-day-old *N. punctiforme* cells were harvested by centrifugation at 4,000 rpm for 5 min at room temperature and washed twice with PBS (0.01 M, pH 7.4). After thoroughly removing all PBS, cells were weighed and resuspended in 1.5 ml of methanol/ $CHCl_3$ (2:1 v/ v). Next, internal standards were added (500 ng of d17:0/ d17:1 and 2,000 ng of d17:1-P). The suspension was then vortexed vigorously for 1 minute before probe-sonicating at 20 kHz for 1 minute 30 sec, in 30 second bursts. Once homogenised, the cell suspension was transferred to a 7 ml glass vial and incubated overnight at 48°C. The next day, cells were cooled on ice before adding 150 µl 1 M KOH. Next, cells were vortexed as before and sonicated in a sonication bath at 40 kHz for 10 min before incubating at 37°C for 2 hr. After cooling on ice, the sample was neutralised with acetic acid and centrifuged at 3,800 rpm for 8 min. From this, the organic supernatant was transferred to a new vial and

the cell debris retained for subsequent extraction. To the cell pellet, 1 ml of methanol/ $CHCl_3$ (1:2 v/ v) before vortexing for 1 minute and centrifuging at 3,800 rpm for 8 min. From this, the organic supernatant was combined with the previous one. The cell pellet was then extracted a third time by dissolving in 0.4 ml of methanol/ $CHCl_3$ (2:1 v/ v), 1 ml of $CHCl_3$ and 2 ml water. After vortexing for 15 sec, cell debris was collected once again by centrifugation at 3,800 rpm for 8 min. From this, the organic phase was combined with the previous ones, whilst 1 ml of $CHCl_3$ was added to the aqueous phase for a fourth and final extraction. Once added, the sample was centrifuged as before and the organic phase once again combined with all of the previous ones. Finally, the pooled organic phases were dried under a nitrogen stream and stored at -20°C for later analysis.

4) "Markham" method [5]

This is a lipid extraction method designed to enrich for sphingolipids and proved the most effective for extracting sphingolipids in this work. It is based on "Method IV" from Markham *et al*. (*5*) and is depicted in Fig. S1. This method was successfully used in this work as follows. *N. punctiforme* cells were harvested 21 days after inoculating 100 ml of either $BG11_0(NH_4)$ or $BG11_0$ with a volume of cells equivalent to 60 µg chlorophyll *a*. To achieve a sufficient biomass, 6 × 100 ml cultures (*ca.* 500 mg) were pooled in a single 50 ml tube (centrifuged at 3,000 *g* for 5 min). Once pooled, cells were transferred to a 2 ml Eppendorf™ tube and centrifuged at 20,000 *g* for 3 min to thoroughly remove all culture media by pipetting. After pelleting, cells were weighed, flash-frozen in liquid N_2 and transferred to a 7 ml glass vial with a PFTE-faced rubber-lined lid (Sigma-Aldrich®; ref. Z106437). Once transferred, cells were immediately re-suspended in 4 ml of (isopropanol/ n-hexane/ H_2O (55:20:25 v/ v/ v) and vortexed vigorously for 1 minute. As positive control, simultaneously, 120 mg of lyophilised, 7-day-old *A. thaliana* cells were extracted. These had been prepared previously by centrifuging a liquid cell suspension culture at 4,400 *g* and washed twice with 0.01 M phosphate-buffered saline (PBS; pH = 7.4) to remove sugars, after which cells were flash-frozen and freeze-dried. A blank extract was also prepared using identical method except that no biological material was added to the extraction solvent. To add internal standards, volumes of individual LCB d17:0, d17:1 and d17:1-P corresponding to 3, 4 and 5 µg, respectively, were combined in a 1.5 ml LC-MS vial (Agilent©;

ref.5182-0715) – one per extraction/ sample. The solvent was evaporated by a gentle stream of nitrogen and the standards were dissolved in 500 µl of extraction solvent. The pooled standard was then transferred to the sample vial. Next, another 500 µl of extraction solvent was used to rinse the vials in order to maximise the transferal of the initial amount of internal standards prepared, bringing the total extraction volume to 5 ml. This cells suspension was then homogenised at 40 kHz in an ice-cold sonication bath for 30 min. After homogenisation, samples were incubated for 15 min at 60°C with intermittent vortexing. Whilst still warm, samples were centrifuged at 500 *g* for 10 min at room temperature. From this, the organic supernatant was transferred to a new vial and placed under a nitrogen stream for drying. In the meantime, the pellet was extracted once more with 5 ml of extraction solvent and incubated for 15 min at 60°C, as before. After centrifugation, the decanted second supernatant was added to the previous vial and dried as before. As the extraction solvent is 25% water, it was not feasible to completely dry samples using the nitrogen stream. Therefore, the remaining water phase was promptly frozen in liquid nitrogen and placed in a freeze dryer for overnight sublimation. The next day, the dried crude extracts, which contained a sizeable amount of solid material, were kept at -20°C until LC-MS analysis.

Calculation of sphingolipid recovery, sample matrix effects and process efficiency.

To estimate the level of sphingolipid loss during the extraction process the recovery (*R*) levels of internal standards were calculated according to the following equation:

$$R(\%) = \frac{Pre^{\mathrm{PA}}}{Post^{\mathrm{PA}}} \times 100$$

where the peak area (PA) for an individual standard is measured before (*Pre*) and after (*Post*) spiking a known concentration of internal standards (2 µg per sample extract). To estimate the levels of sphingolipids that were sequestered in the insoluble sample debris, sample matrix effects (*M*) were calculated using the following equation:

$$M(\%) = \frac{Pre^{\mathrm{PA}}}{St.sol^{\mathrm{PA}}} \times 100$$

where the Pre^{PA} is measured as before, and the PA of the standard solvent (*St.sol*) is measured by directly injecting an equivalent amount of the internal standard mix (*i.e*., 3, 4 and 5 µg combined). Using the *R* and *M* values, the overall process efficiency (*P*) can be calculated using the following equation:

$$P(\%) = \frac{M \times R}{100}$$

This final measure should theoretically indicate the total sphingolipid amount captured during the extraction process.

$$P(\%) = \frac{M \times R}{100}$$

1. **Bligh EG, Dyer WJ**. A rapid method of total lipid extraction and purification. *Can J Biochem Physiol* 1959;37:911–7.

2. **Folch J, Lees M, Sloane Stanley GH**. A simple method for the isolation and purification of total lipides from animal tissues. *J Biol Chem* 1957;226:497–509.

3. **Merrill AH Jr, Sullards MC, Allegood JC, Kelly S, Wang E**. Sphingolipidomics: high-throughput, structure-specific, and quantitative analysis of sphingolipids by liquid chromatography tandem mass spectrometry. *Methods* 2005;36:207–24.

4. **Wang JR, Zhang H, Yau LF, Mi JN, Lee S, *et al.*** Improved sphingolipidomic approach based on ultra-high performance liquid chromatography and multiple mass spectrometries with application to cellular neurotoxicity. *Anal Chem* 2014;86:5688–96.

5. **Markham JE, Li J, Cahoon EB, Jaworski JG**. Separation and identification of major plant sphingolipid classes from leaves. *J Biol Chem* 2006;281:22684–94.

Table S1. Recovery levels of C17 sphingolipid standards in lipid extracts prepared from *Arabidopsis thaliana* and *Nostoc punctiforme* cell samples. Different lipid extraction methods, which are indicated in the left-most column, yielded varying standard recovery levels. The “Markham” method – described as “method IV” in Markham *et al*. [5] – proved to be the most suitable for extracting lipids from *N. punctiforme*. *n* = 1.

	A. thaliana			***N. punctiforme***		
	**d17:0**	**d17:1**	**d17:1-P**	**d17:0**	**d17:1**	**d17:1-P**
**Bligh and Dyer**	20%	24%	5%	3%	5%	0%
**Folch**	-	-	-	-	-	-
**Merrill**	8%	11%	2%	5%	5%	0%
**Markham**	14%	21%	10%	38%	13%	14%

Table S2. List of sphingolipid standards used in this work. C17 sphingolipids were used as internal standards during lipid extraction and were used to calculate recovery levels, matrix effects and overall process efficiency. All listed sphingolipids were used as references of retention times of natural sphingolipids and to generate standard curves for instrument calibration and natural sphingolipid quantification.

**Common name**	**Shorthand designation**	**Molecular formula**	**Dissolved in**	**Source**
C16 sphingosine	d16:1	$C_{16}H_{33}NO_2$	Methanol	Avanti®; ref no. 860669
C17 sphinganine	d17:0	$C_{17}H_{37}NO_2$	Methanol	Avanti®; ref no. 860654
C17 sphingosine	d17:1	$C_{17}H_{35}NO_2$	Methanol	Avanti®; ref no. 860640
C17 sphingosine-1-phosphate	d17:1-P	$C_{17}H_{36}NO_5P$	Methanol/ H_2O (3:1, v/ v)	Avanti®; ref no. 860641
C18 sphinganine	d18:0	$C_{18}H_{39}NO_2$	Methanol	Avanti®; ref no. 860498
C18 sphingosine	d18:1	$C_{18}H_{37}NO_2$	Methanol	Avanti®; ref no. 860490
C18 sphinganine-1-phosphate	d18:0-P	$C_{18}H_{41}NO_5P$	Methanol	Avanti®; ref no. 860536
C18 sphingosine-1-phosphate	d18:1-P	$C_{18}H_{39}NO_5P$	Methanol	Avanti®; ref no. 860492
C18 phytosphingosine	t18:0	$C_{18}H_{39}NO_3$	Methanol	Matreya LLC© ref no. 1330
C12 Ceramide	Cer(d18:1/ 12:0)	$C_{30}H_{59}NO_3$	Ethanol	Avanti®; ref no. LM6002
C12 Ceramide-1-phosphate	CerP(d18:1/ 12:0)	$C_{30}H_{60}NO_6P$	Ethanol	Avanti®; ref no. LM6002
C12 Glucosylceramide	CerGluc(d18:1/ 12:0)	$C_{36}H_{70}NO_8$	Ethanol	Avanti®; ref no. LM6002
C12 Lactosylceramide	CerLac(d18:1/ 12:0)	$C_{42}H_{80}NO_{13}$	Ethanol	Avanti®; ref no. LM6002
C25 Ceramide	Cer (d18:1/ 25:0)	$C_{43}H_{85}NO_3$	Ethanol	Avanti®; ref no. LM6002

Table S3. Standard sphingolipid reference information. Retention times were derived after UHPLC analysis according to the run conditions described in the methods section of this work. For quantitative analysis of natural sphingolipids, standard (STD) curves were generated (Fig. S2) within the ranges indicated, with the reliability of each curve expressed by the given correlation coefficient (R^2). The limit of detection (LOD) is defined as the concentration during standard curve generation at which abundance (signal intensity) was less than $5x10^2$.

**Sphingolipids**	**Rt (min)**	**Expected *m/z* $[M+H]^+$**	**Measured *m/z***	**ppm error (MS/MS)**	**Expected product ion**	**Measured product ion**	**CE (eV)**	**STD curve ($ng\ ml^{-1}$) From:**	**STD curve ($ng\ ml^{-1}$) To:**	**R^2**	**LOD ($ng\ ml^{-1}$)**
d16:1	4.94	272.2584	272.2578	-2.20	254.2483	254.2480	12	3	300	0.9978	0.3
d17:1	6.06	286.2740	286.2744	1.40	268.2636	268.2700	12	3	300	0.9995	0.3
d17:0	6.63	288.2897	288.2900	1.04	270.2797	270.2800	18	3	300	0.9998	0.1
d17:1-P	5.94	366.2404	366.2409	1.37	250.2522	250.2500	12	30	3000	0.9987	3
d18:1	7.16	300.2897	300.2897	0.00	282.2796	282.2797	12	3	300	0.9943	0.1
d18:1-P	7.16	380.2560	380.2559	-0.26	264.2688	264.2685	12	30	3000	0.9994	3
d18:0	7.77	302.3053	302.3051	-0.66	284.2956	284.2946	18	3	300	0.9932	0.3
d18:0-P	7.76	382.2717	382.2718	0.26	284.3000	284.2949	12	30	3000	0.9994	3
t18:0	6.89	318.3003	318.3002	-0.31	300.3000	300.2896	22	3	300	0.9966	0.1
Cer(d18:1/ 12:0)	16.92	482.4568	482.4573	1.04	264.3000	264.2693	12	3	300	0.9975	1
Cer(d18:1/ 12:0)	14.64	562.4231	562.4214	-3.02	264.3000	264.2684	18	30	3000	0.9989	3
GlcCer(d18:1/ 12:0)	15.78	644.5096	644.5077	-2.95	264.3000	264.2686	12	3	300	1.0000	1
LacCer(d18:1/ 12:0)	15.26	806.5624	806.5616	-0.99	264.3000	264.2687	12	3	300	0.9992	1
Cer(d18:1/ 25:0)	22.31	664.6602	664.6600	-0.30	264.3000	264.2689	12	3	300	0.9993	1

Table S4. Reference list of LCBs and ceramides used for mining for sphingolipids in mass spectra of EICs generated by UHPLC/ Q-TOF-MS analysis of *Nostoc punctiforme* and *Arabidopsis thaliana* cell lipid extracts.

	Molecular Formula	Monoisotopic mass	$[M+H]^+$	Product ion	MS/MS targeted
**LCB (C14)**					
d14:1	$C_{14}H_{29}NO_2$	243.2200	244.2273	226.2120	Yes
**LCB (C16)**					
d16:0	$C_{16}H_{35}NO_2$	273.2668	274.2740	256.2590	Yes
d16:1	$C_{16}H_{33}NO_2$	271.2511	272.2584	254.2434	Yes
t16:0	$C_{16}H_{35}NO_3$	289.2617	290.2690	272.2540	No
t16:1	$C_{16}H_{33}NO_3$	287.2460	288.2533	270.2383	No
d16:0-P	$C_{16}H_{36}NO_5P$	353.2331	354.2404	336.2254	Yes
d16:1-P	$C_{16}H_{34}NO_5P$	351.2175	352.2248	335.2097	Yes
t16:0-P	$C_{18}H_{36}NO_6P$	369.2280	370.2353	352.2203	No
t16:1-P	$C_{18}H_{34}NO_6P$	367.2306	368.2379	350.2229	No
**LCB (C18)**					
d18:0	$C_{18}H_{39}NO_2$	301.2981	302.3053	284.2903	Yes
d18:1	$C_{18}H_{37}NO_2$	299.2824	300.2897	282.2747	Yes
t18:0	$C_{18}H_{39}NO_3$	317.2930	318.3003	300.2896	Yes
t18:1	$C_{18}H_{37}NO_3$	315.2773	316.2846	298.2696	Yes
d18:0-P	$C_{18}H_{41}NO_5P$	381.2644	382.2717	266.3	Yes
d18:1-P	$C_{18}H_{39}NO_5P$	379.2488	380.2560	264.3	Yes
t18:0-P	$C_{18}H_{41}NO_6P$	397.2593	398.2666	300.3	Yes
t18:1-P	$C_{18}H_{38}NO_6P$	395.2437	396.2150	298.3	Yes
**LCB (C20)**					
d20:0	$C_{20}H_{43}NO_2$	329.3293	330.3366	312.3216	Yes
d20:1	$C_{20}H_{41}NO_2$	327.3137	328.3210	310.3060	Yes
t20:0	$C_{20}H_{43}NO_3$	345.3243	346.3316	328.3166	No
t20:1	$C_{20}H_{41}NO_3$	343.3086	344.3159	326.3009	No
d20:0-P	$C_{20}H_{44}NO_5P$	409.2957	410.3030	392.2880	No
d20:1-P	$C_{20}H_{42}NO_6P$	407.2801	408.2874	390.2724	No
t20:0-P	$C_{20}H_{44}NO_5P$	425.2906	426.2979	408.2829	No
t20:1-P	$C_{20}H_{42}NO_6P$	423.2750	424.2823	406.2673	No
**Cer (C18)**					
Cer(d18:1/ 12:0)	$C_{34}H_{69}NO_3$	481.7940	482.4568	264.3	Yes
CerP(d18:1/ 12:0)	$C_{30}H_{60}NO_6P$	561.4158	562.4231	264.3	Yes
Cer(d18:0/ 16:0)	$C_{34}H_{69}NO_3$	539.5277	540.5350	266.3	Yes
Cer(d18:0/ 16:0)	$C_{34}H_{67}NO_3$	537.5121	538.5194	264.3	Yes
Cer(d18:0/ 18:0)	$C_{36}H_{73}NO_3$	567.5590	568.5663	266.5	Yes
Cer(d18:1/ 18:0)	$C_{36}H_{71}NO_3$	565.5434	566.5507	264.5	Yes
Cer(d18:1/ 25:0)	$C_{43}H_{85}NO_3$	663.6529	664.6602	264.3	Yes
Cer(t18:0/ 16:0)	$C_{34}H_{69}NO_4$	555.5227	556.5300	300.3	No
Cer(t18:0/ 18:0)	$C_{36}H_{73}NO_4$	583.5540	584.5613	300.3	Yes
Cer(t18:0/ 20:0)	$C_{38}H_{77}NO_4$	611.5853	612.5926	300.3	Yes
Cer(t18:0/ 22:0)	$C_{40}H_{81}NO_4$	639.6166	640.6239	300.3	Yes
Cer(t18:0/ 24:0)	$C_{42}H_{85}NO_4$	667.6479	668.6552	300.3	Yes
Cer(t18:0/ 26:0)	$C_{44}H_{89}NO_4$	695.6792	696.6865	300.3	No
**Cer-OH (C18)**					
Cer(t18:0/ 18:0(2OH))	$C_{36}H_{73}NO_5$	599.5489	600.5562	300.3	Yes
Cer(t18:0/ 20:0(2OH))	$C_{38}H_{77}NO_5$	627.5802	628.5875	300.3	Yes
Cer(t18:1(8Z)/ 20:0(2OH))	$C_{38}H_{75}NO_5$	625.5645	626.5718	298.3	Yes
Cer(t18:0/ 22:0(2OH))	$C_{40}H_{81}NO_5$	655.6115	656.6188	300.3	Yes
Cer(t18:0)/ 24:0(2OH))	$C_{42}H_{85}NO_5$	683.6428	684.6501	300.3	Yes
Cer(t18:0/ 26:0(2OH))	$C_{44}H_{89}NO_5$	711.6741	712.6814	300.3	Yes
Cer(t18:1(8E)/ 26:0(2OH))	$C_{44}H_{87}NO_5$	709.6584	710.6657	298.3	Yes
**Cer-Glu (C18)**					
GlcCer(d18:1/12:0)	$C_{36}H_{70}NO_8$	643.5023	644.5096	264.3	Yes
GlcCer(t18:1(8Z)/ 20:0(2OH[S]))	$C_{44}H_{85}NO_{10}$	787.6173	788.6246	298.3	Yes
GlcCer(t18:1(8Z)/ 22:0(2OH[S]))	$C_{46}H_{89}NO_{10}$	815.6486	816.6559	298.3	Yes
GlcCer(t18:1(8E)/ 24:0(2OH[R]))	$C_{48}H_{93}NO_{10}$	843.6799	844.6872	298.3	Yes
GlcCer(t18:1(8Z)/ 26:0(2OH[R]))	$C_{50}H_{97}NO_{10}$	871.7112	872.7185	298.3	Yes
**Cer-Lac (C18)**					
LacCer(d18:1/ 12:0)	$C_{42}H_{80}NO_{13}$	805.5551	806.5624	264.3	Yes

Table S5. C18 long chain base (LCB) and ceramide sphingolipids identified by UHPLC/ Q-TOF-MS/ MS analysis of 7-day-old *Arabidopsis thaliana* Ler-0 cells grown in liquid suspension culture. Measured masses (*m/z*) were recorded within a mass accuracy error of 5 ppm from the monoisotopic mass in positive polarity $[M+H]^+$. Additional indicated parameters include retention time (RT), collision energy (CE), average mass error (E) and the number (n) of biological replicates (Rep) in which each sphingolipid was detected.

	**Molecular formula**	**Monoisotopic mass**	**$[M+H]^+$**	**Measured *m/z***	**E ($\bar{x}$ ppm) ± S.D**		**RT (min)**	**CE (eV)**	**Rep (*n*)**
**LCBs (C18)**									
d18:0	$C_{18}H_{39}NO_2$	301.2981	302.3053	302.3060	2.23 ± 0.83	284.2950	7.746	5	4
t18:0	$C_{18}H_{39}NO_3$	317.2930	318.3003	318.3000	1.81 ± 1.94	300.2894	6.837	12	4
t18:1	$C_{18}H_{37}NO_3$	315.2773	316.2846	316.2855	3.32 ± 0.75	298.2738	5.889	12	4
**Ceramides (C18)**									
Cer(d18:0/ 16:0)	$C_{34}H_{69}NO_3$	539.5277	540.5350	540.5367	3.24 ± 0.75	266.2833	19.283	18	4
Cer(d18:1/ 16:0)	$C_{34}H_{67}NO_3$	537.5121	538.5194	538.5211	1.25 ± 1.61	264.2691	18.458	22	4
Cer(t18:0/ 20:0)	$C_{38}H_{77}NO_4$	611.5853	612.5926	612.5894	0.69 ± 1.13	300.2767	20.237	18	3
Cer(t18:0/ 22:0)	$C_{40}H_{81}NO_4$	639.6166	640.6239	640.6262	1.40 ± 1.79	300.2873	20.917	12	4
Cer(t18:0/ 24:0)	$C_{42}H_{85}NO_4$	667.6479	668.6552	668.6552	2.24 ± 1.96	300.2910	21.669	12	4
Cer(t18:1(8Z)/ 20:0(2OH))	$C_{38}H_{75}NO_5$	625.5645	626.5718	626.5733	1.39 ± 1.47	298.2736	18.809	12	2
Cer(t18:0/ 22:0(2OH))	$C_{40}H_{81}NO_5$	655.6155	656.6188	656.6205	1.10 ± 1.13	300.2960	20.633	5	4
Cer(t18:0) /24:0(2OH))	$C_{42}H_{85}NO_5$	683.6428	684.6501	684.6503	1.61 ± 1.61	300.2950	21.355	18	4

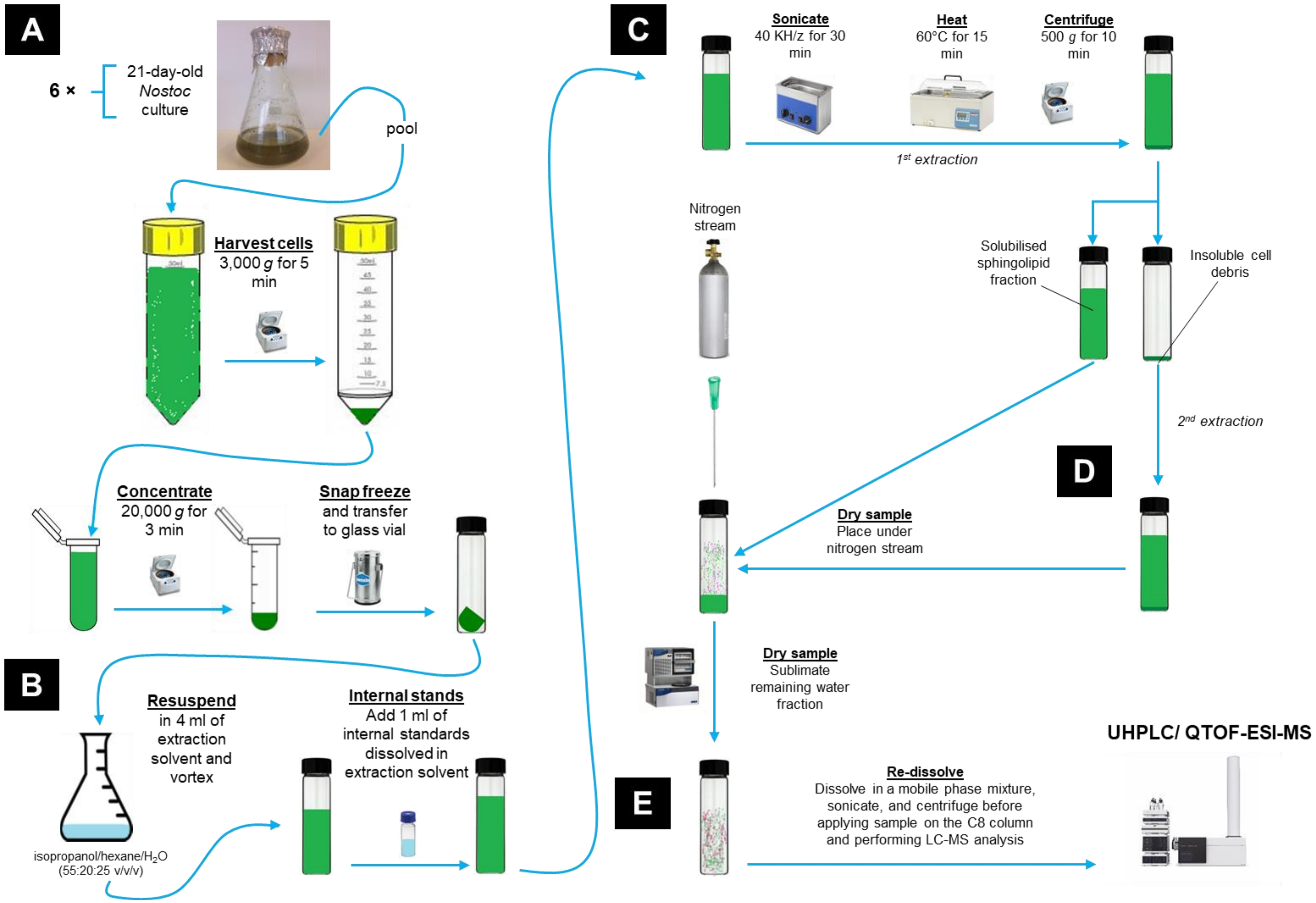

Fig. S1. Overview of sphingolipid extraction method adapted from Markham *et al*. [5]. **(A)** Six mature *Nostoc punctiforme* cultures (ca. 500 mg) are pooled and concentrated in a single 2 ml Eppendorf™ tube before flash-freezing in liquid N_2 and transferring to a 7 ml glass vial. **(B)** The frozen cell pellet is resuspended in isopropanol/ n-hexane/ H_2O before adding internal standards. **(C)** Cells are homogenised and membranes melted during a heat incubation step (releasing membrane-bound sphingolipids). After pelleting the insoluble cell debris **(D)** the soluble organic fraction is placed under a nitrogen stream for drying, whereas the pellet is extracted once more. Supernatants are then combined for further drying down to the water component, at which point the sample is flash frozen and placed in a freeze dryer for overnight sublimation. **(E)** Once dry, extracts are thoroughly re-dissolved in a mixture of mobile phases (500 µl of isopropanol/ methanol (2:1 v/ v) and 500 µl of H_2O/ methanol (2:1 v/ v)), sonicated, centrifuged and analysed by UHPLC/ QTOF-ESI-MS.

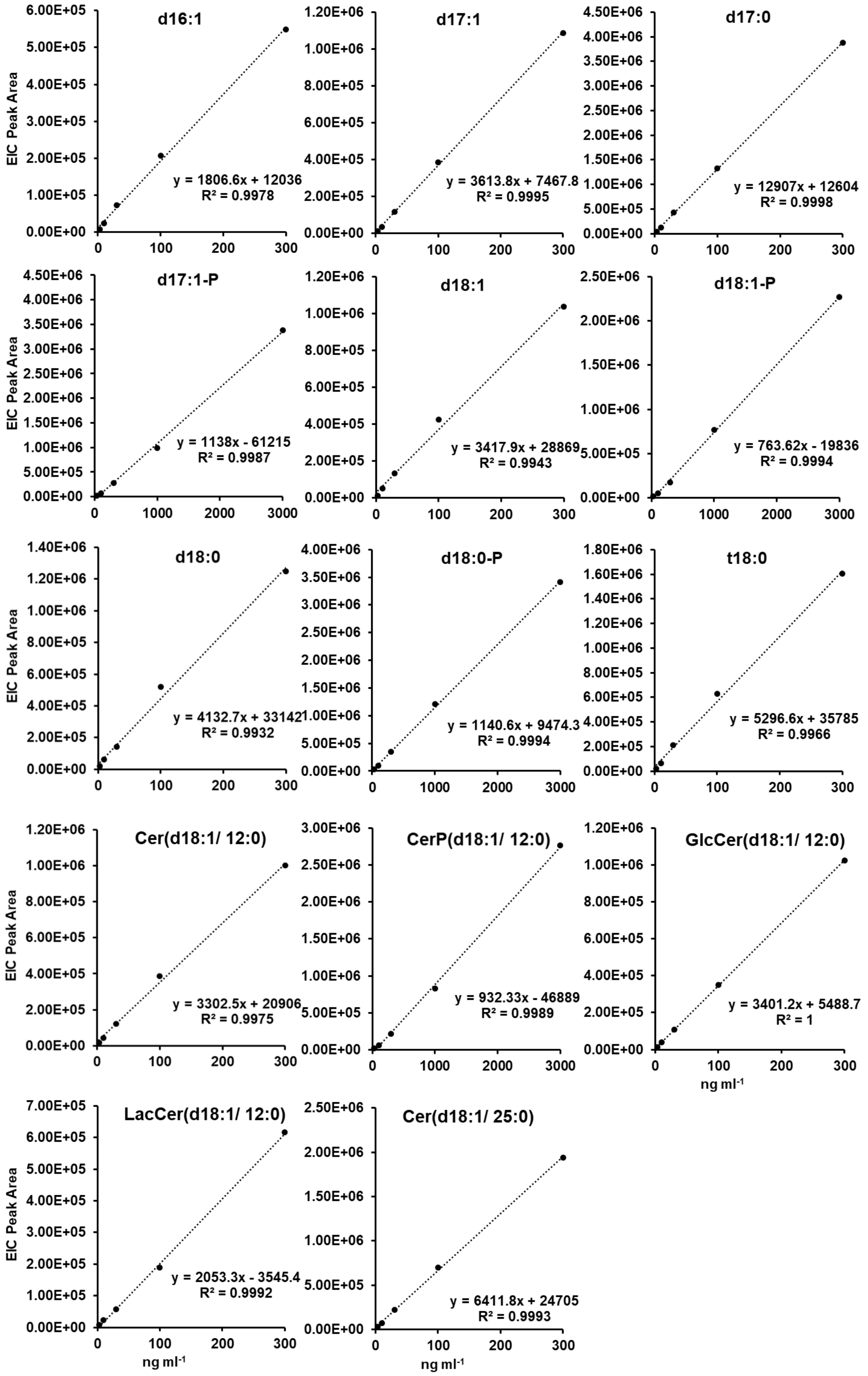

Fig. S2. LCB (C16, C17 and C18) and ceramide sphingolipid standard curves used for quantitative analysis of natural sphingolipids in this work.

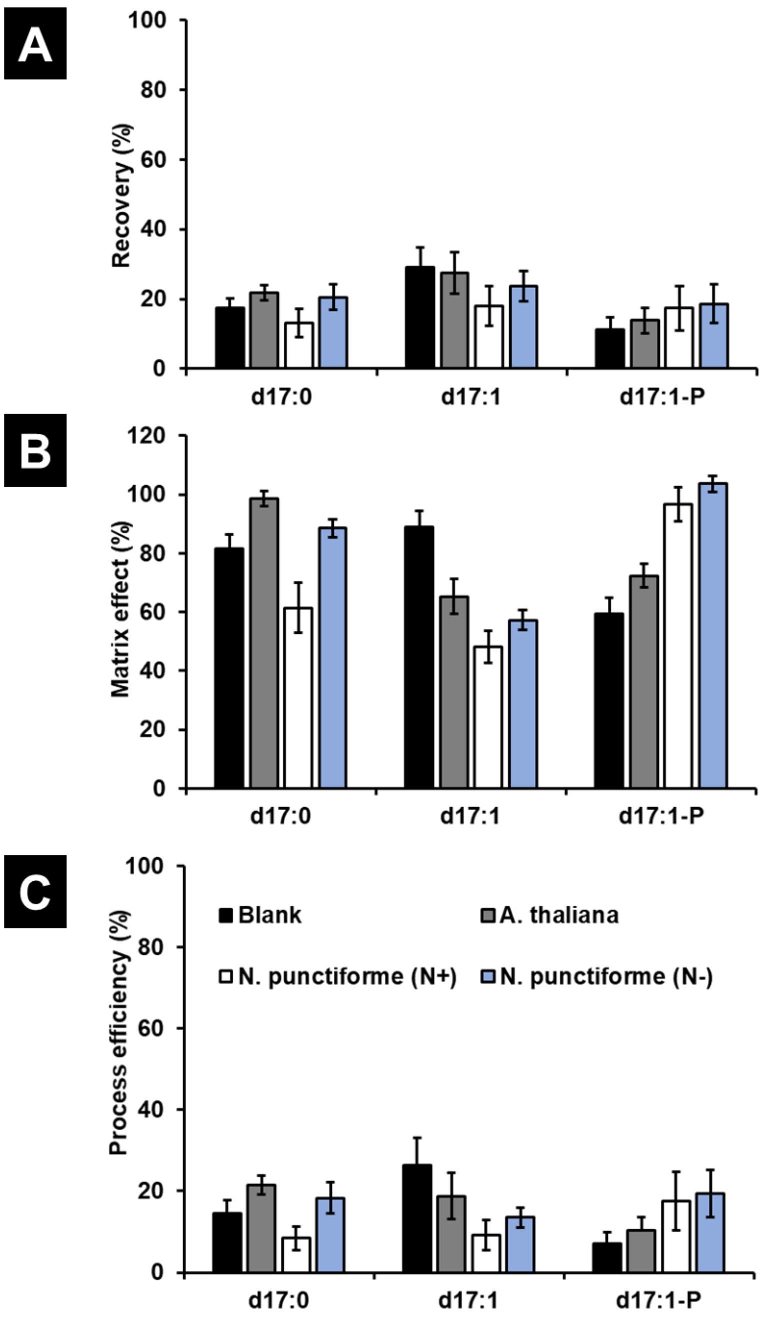

Fig. S3. Three estimations of sphingolipid extraction efficiency were calculated based on three different internal standards; d17:0, d17:1 and d17:1-P. **(A)** Recovery (%) values indicate sphingolipid loss during the extraction process. **(B)** The matrix effect (%) indicates the effects of sample biomass on sphingolipid extraction. When this measure is >100%, ionisation enhancement has taken place as a result of sample compounds coeluting. When <100%, a matrix effect has occurred. **(C)** Once the recovery levels and matrix effects have been determined, the overall process efficiency (%) can then be calculated. Samples include a no-biomass extraction (Blank), *Arabidopsis thaliana* and *Nostoc punctiforme* grown with (N+) or without (N-) a source of combined nitrogen (ammonium). Values are means ± S.E., n = 4.

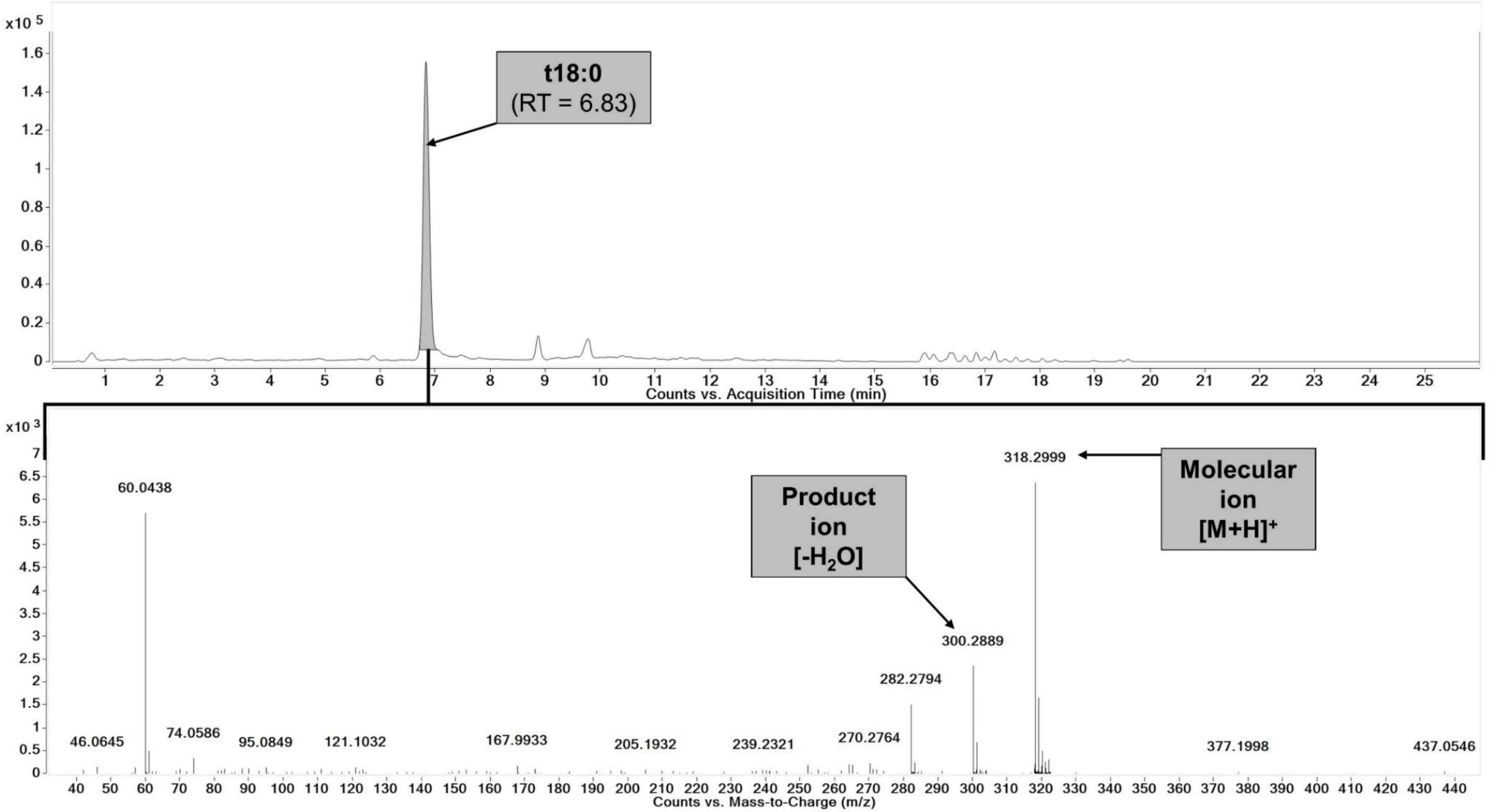

Fig. S4. Identification by UHPLC/ Q-TOF-MS/ MS analysis of t18:0 (phytosphingosine) in lipids extracted from 7-day-old *Arabidopsis thaliana* (Ler-0) cells grown in liquid suspension culture. Top panel shows the EIC of t18:0 at a retention time (RT) of 6.83 min. Bottom panel shows the corresponding MS/ MS spectrum depicting the molecular ion and its diagnostic product ion derived by CID at 12 eV.

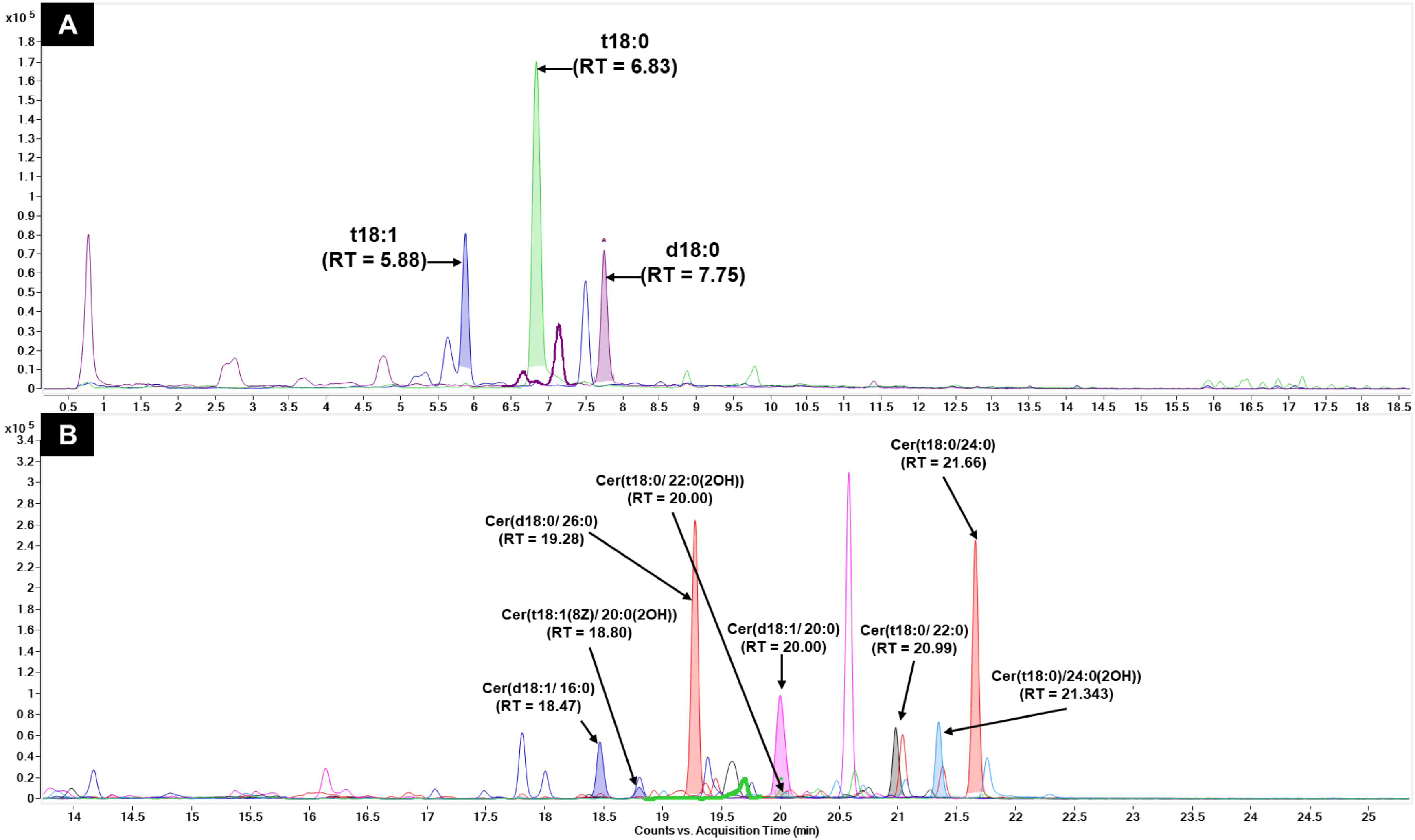

Fig. S5. Identification by UHPLC/ Q-TOF-MS analysis of LCB **(A)** and ceramide **(B)** species in lipid extracts from 7-day-old Arabidopsis thaliana Ler-0 cells. Shown are the merged extracted ion chromatograms depicting each species at their respective retention times (RT).

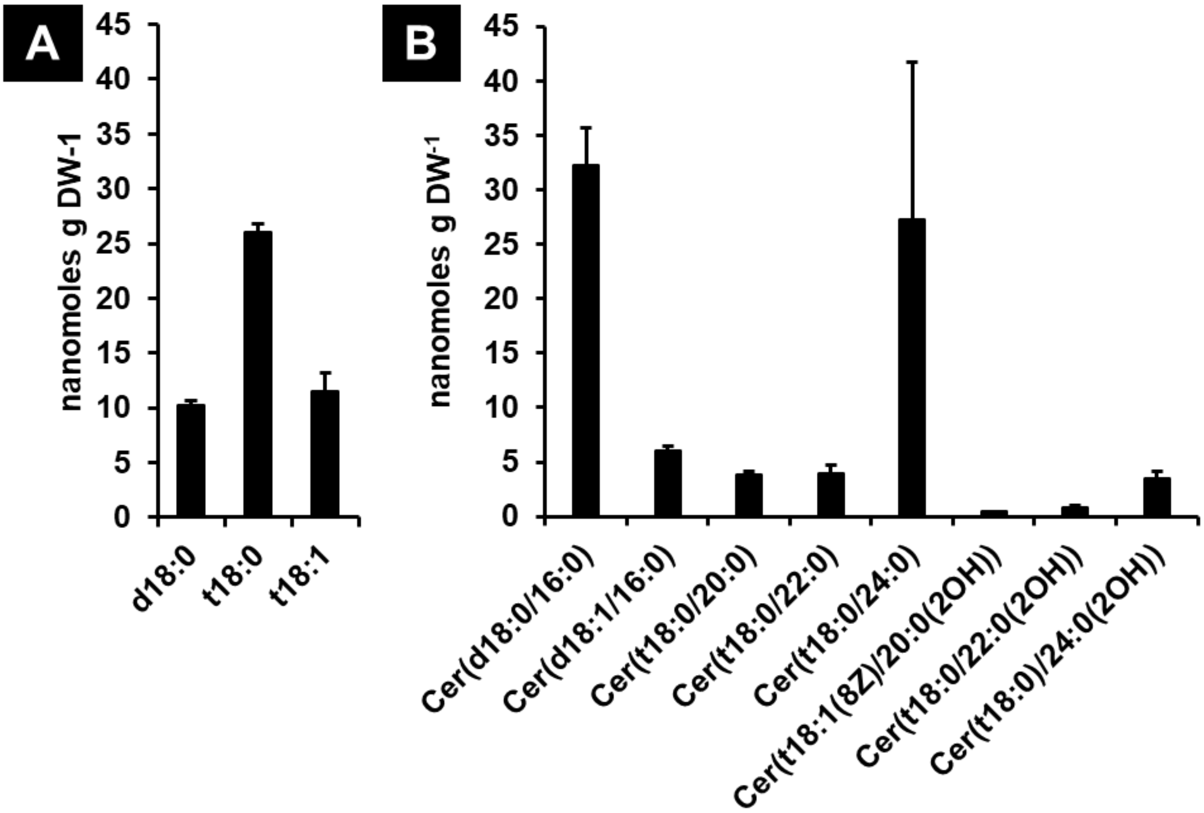

Fig. S6. Measurement of selected **(A)** LCB and **(B)** complex sphingolipids in *Arabidopsis thaliana* (Ler-0) cells derived from liquid suspension cultures. Sphingolipid amounts are expressed as nanomoles g dry weight (DW)$^{-1}$ and were quantified using the following standard curves: d18:0 was quantified using a d18:0 standard curve, whereas t18:0 and t18:1 were quantified using a t18:0 standard curve. Simple ceramides were quantified using C12 and C25 ceramide standard curves, whereas hydroxylated ceramides were quantified using a C12 glucosylceramide standard curve. Only sphingolipids which could be annotated by MS/ MS analysis in at least 2 biological replicates were considered for quantification. Values are means ± S.E, *n* = 4.

Supplementary Data Sheet 2

Table S6. Primer sequences used for RT-qPCR analysis of *Npun_R3567*. An annealing temperature of 60°C was used for all primers. Results of primer testing are shown in Fig. S9 and Fig. S10.

Primer	Sequence (5'-3')	Application
Npun_F5020-1<br>Npun_F5020-2	CTGCGGGAATCGGAAAAAGA<br>CCAATCGGTAGGCTGAGAAATAAT	RT-qPCR; Housekeeping gene
Npun_F5466-1<br>Npun_F5466-2	CCGTAGCAGTTAATGGTGGAC<br>TGTAAGCAGTTTGTGGGGATAAG	RT-qPCR; Housekeeping gene
Npun_R1722-1<br>Npun_R1722-2	TAGAGCCGAAACGAGTAAAA<br>AATCAAATAGCGAGGTTCTT	RT-qPCR; marker for diazotrophy (*HetR*).
Npun_F2507-1<br>Npun_F2507-2	CTTTAGTACGCAGAGGCAGATGAC<br>AGGTATTGGCGGTGGGCTAAC	RT-qPCR; marker for motility (*PilT*).
Npun_R3567-9<br>Npun_R3567-10	CTGGGGATAAGCGGTTGA<br>AGGCATCTTCTGTTTGTTTGGTA	RT-qPCR; *Npun_R3567.*

Table S7. Frequency (%) of different *Nostoc punctiforme* cell types at 1-, 3-, 5-, 10- and 20-day(s) post-induction (dpi) for diazotrophic growth ($BG11_0$). Values are ±S.E. Significant differences (yellow) between treatments were determined using a Student's T-Test (*p ≤ 0.05, **p ≤ 0.01, ***p ≤ 0.001) on arcsine-transformed proportions. *n* = 5, *ca*. 250-500 cells scored per replicate.

	Vegetative cells		Hormogonia		Heterocysts	
	**$BG11_0(NH_4)$**	**$BG11_0$**	**$BG11_0(NH_4)$**	**$BG11_0$**	**$BG11_0(NH_4)$**	**$BG11_0$**
1 dpi	91.5±5.3	75.3±11.8	8.5±5.3	24.7±11.8	0.0±0.0	0.0±0.0
3 dpi	95.3±2.9	59.1±10.1**	4.7±2.9	39.5±9.4**	0.0±0.0	1.4±1.0
5 dpi	90.0±3.4	51.2±10.5**	9.2±3.3	47.0±11**	0.70±0.6	1.8±0.5
10 dpi	73.9±13.6	94.4±1.0	26.1±13.	0.0±0.0	0.0±0.0	5.6±1.0***
20 dpi	96.7±2.0	94.0±0.9	3.3±2.0	0.0±0.0	0.0±0.0	6.0±0.9***

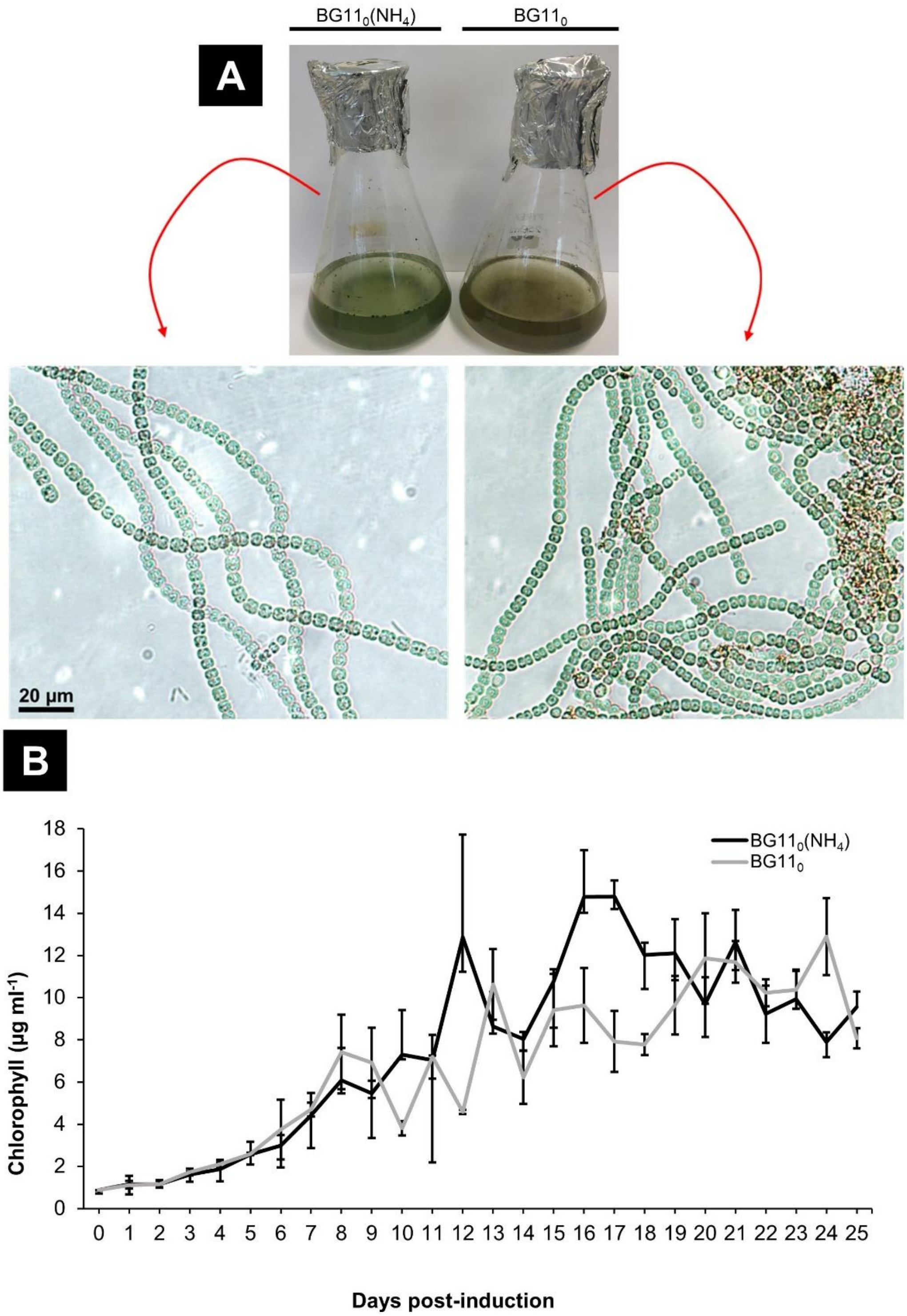

Fig. S7. (A) *Nostoc punctiforme* PCC 73102 liquid cultures 21 days after growth with [BG11$_0$(NH$_4$)] and without (BG11$_0$) a source of combined nitrogen. **(B)** Growth curve plotted using chlorophyll *a* as a proxy for cell density. Values are means ± S.E., n = 5.

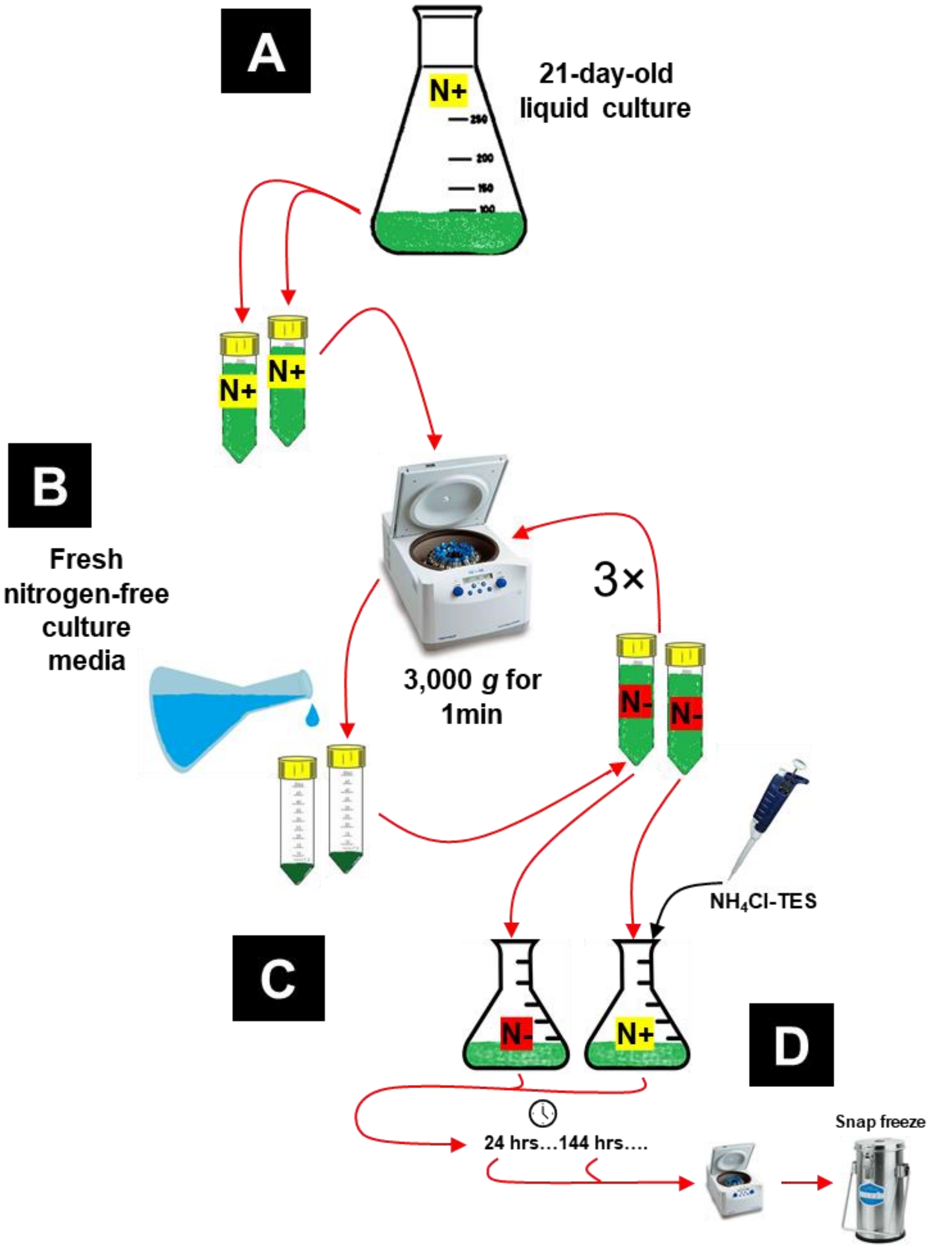

Fig. S8. Overview of workflow for induction of diazotrophy in liquid *Nostoc punctiforme* cultures. **(A)** A 100 ml, 21-day-old, nitrogen-supplemented [$BG11_0(NH_4)$; N+] liquid culture is split between two 50 ml centrifuge tubes. **(B)** These are then centrifuged at 3,000 *g* and re-suspended in fresh, nitrogen-free growth media. This is repeated two more times to completely remove the added combined nitrogen (NH_4Cl-TES) from the starter culture. **(C)** The now-nitrogen-free cultures are then transferred to 50 ml conical flasks before supplementing one with fresh NH_4 (NH_4Cl-TES). **(D)** Cultures are then centrifuged and the pelleted cells flash-frozen in liquid N_2 for subsequent RNA isolation after at least 24 hr of incubation under normal growth conditions.

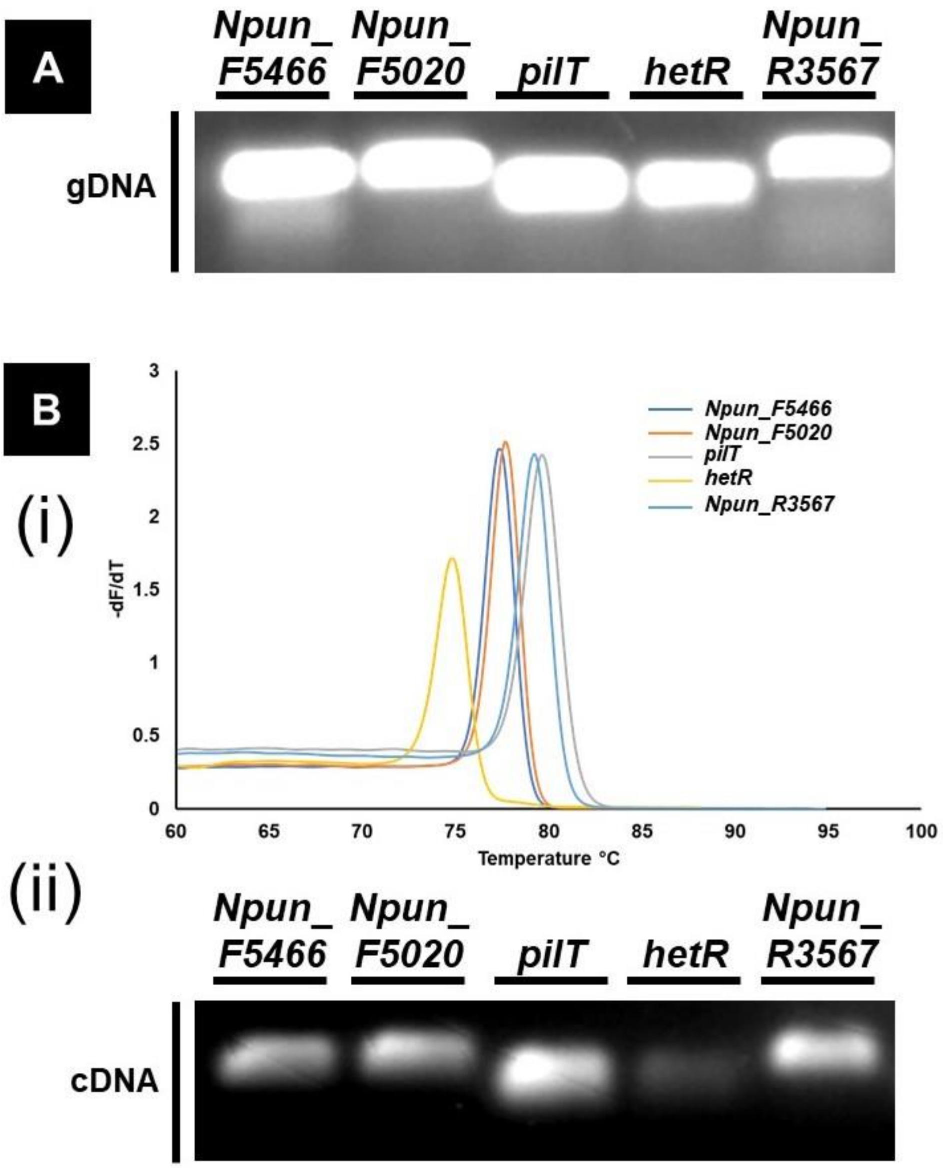

Fig. S9. Testing of RT-qPCR primers used in this work. **(A)** At an annealing temperature of 60°C, primers yielded a single reaction product using a gDNA template. **(B)** (i) Melt curve analysis of RT-qPCR reaction products derived from a cDNA template indicated a single product for each primer pair. (ii) This was confirmed by gel electrophoresis of each RT-qPCR reaction product. Primer amplification efficiencies are shown in Fig. S10.

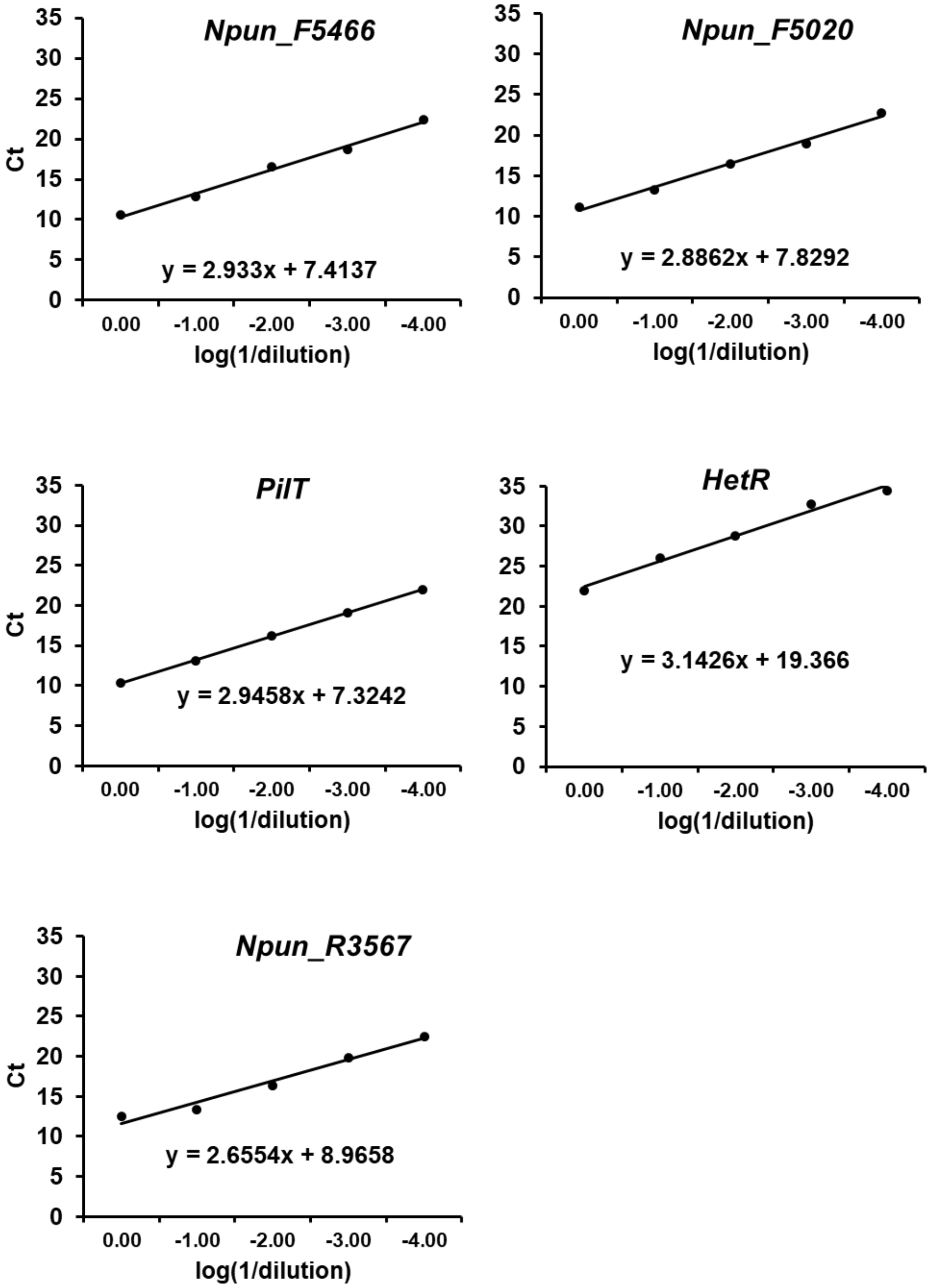

Fig. S10. Standard curves used to calculate amplification efficiencies (*E*) of RT-qPCR primers used in differential gene expression analysis. Shown are the results of RT-qPCR amplification using serial dilutions of gDNA from *Nostoc punctiforme* PCC 73102. *E* was calculated using: $E = 10^{-1}$/m.

Supplementary Data Sheet 3

Table S8. Primer sequences used to PCR-amplify *Npun_R3567* for cloning the into pET21a+ bacterial expression plasmid. Highlighted are BamHI (red) and XhoI (blue) restriction enzymes. Annealing temperature used was 60°C. Target homology is shown in bold.

Primer	Sequence (5'-3')
NpSPTFw7	CCCGGATCC**ATGAATTTTGAATTCGGAGCGA**
NpSPTRv7	AGGCTCGAG**AATAACATCCTTCAGCGCT**

Table S9. Plasmids used to heterologously express *Npun_R3567* and *SmSpt* in *E. coli*.

**Plasmid name**	**Selection in *E. coli***	**Comments**
pET21a+	Ampicillin (50 µg ml^{-1})	Bacterial expression plasmid used to heterologously express *Npun_R3567* and *SmSpt* in *E. coli.*
pEX-K4	Kanamycin (50 µg ml^{-1})	Customised plasmid harbouring *SmSpt* from Eurofins Genomics™.
pUDSB1	Ampicillin (50 µg ml^{-1})	pET21a+ harbouring *Npun_R3567t.*
pUDSB2	Ampicillin (50 µg ml^{-1})	pET21a+ harbouring *SmSpt.*

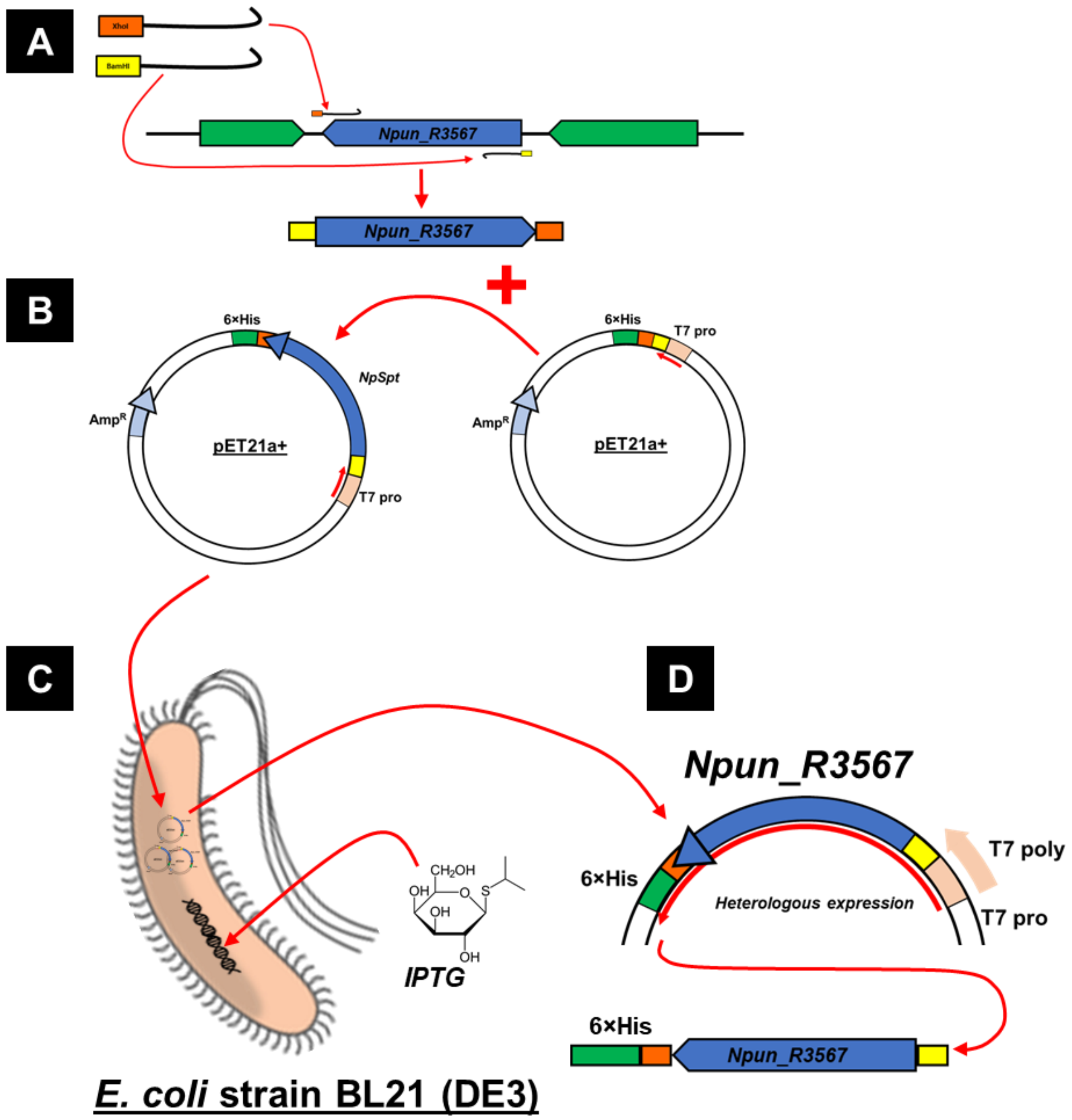

Fig. S11. Overview of strategy used to clone *Npun_R3567* for heterologous expression. **(A)** Forward and reverse introduce restrictions sites BamHI (yellow) and XhoI (orange) at the predicted transcriptional start site and termination site (stop codon removed), respectively. **(B)** The protein expression plasmid pET21a+ is digested with the corresponding restriction enzymes and the recombinant PCR fragment is ligated into the multiple cloning site, just upstream of a polyhistidine tag (6×His). **(C)** The recombinant plasmid is then transformed into the *E. coli* lysogen BL21 (DE3). **(D)** Heterologous expression is induced by adding Isopropyl β-D-1-thiogalactopyranoside (IPTG). This promotes expression of the chromosomally located T7 polymerase (T7 poly), which binds the T7 promoter (T7 pro) immediately upstream of the gene insert.

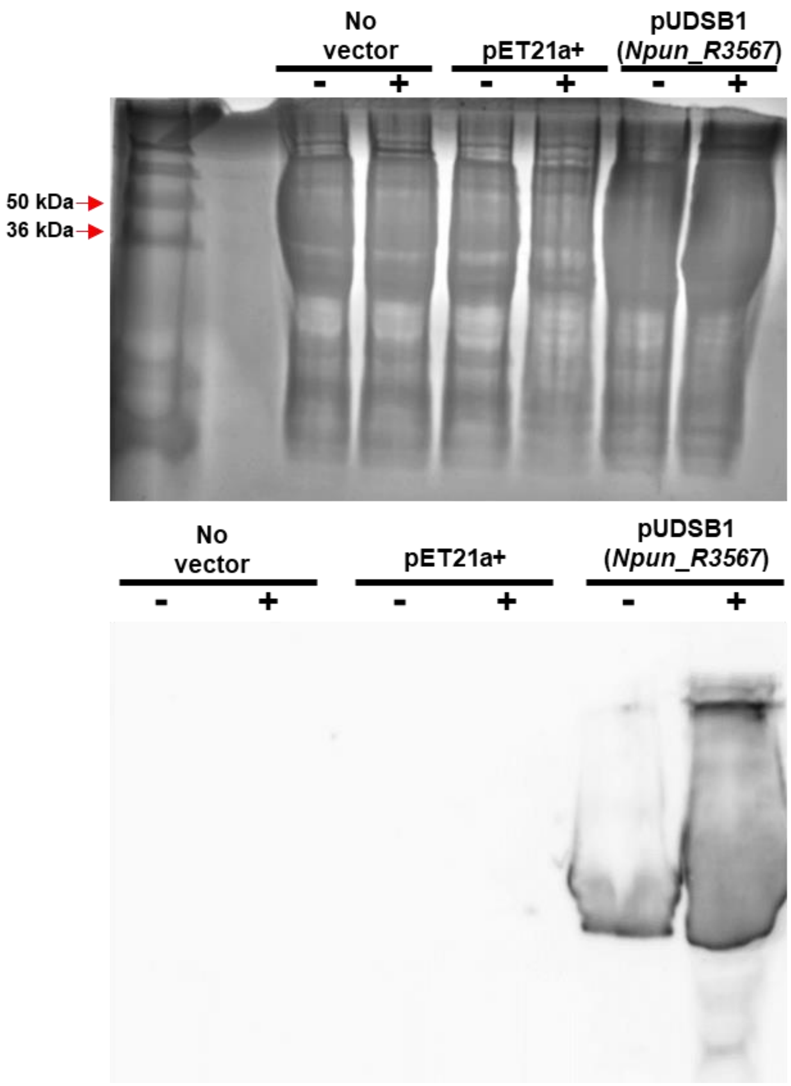

Fig. S12. (Top) Coomassie blue staining of the total crude cellular fraction of *E. coli* BL21(DE3) heterologously expressing the recombinant Npun_R3567. **(Bottom)** Immunoblot detection of recombinant Npun_R3567 in the same cell protein fraction.

Supplementary Data Sheet 4

Table S10. Protein sequences used to infer phylogeny of bacterial POAS enzymes. Accession numbers marked with an asterisk were taken from a phylogeny inferred by Geiger *et al*. [1]. Rows in bold indicate that the sequence has been enzymatically characterised, with references given next to the respective accession numbers.

Species	Accession number	Annotation
Acaryochloris marina	AM1_2296	BioF
Anabaena spp. PCC 7120	all0374	BioF
Aphanothece sacrum	WP_124972400.1	BioF
Calothrix spp. CSU_2_0	NJR16617.1	BioF
Chroococcidiopsis spp. PCC 6712	WP_169243225.1	BioF
Chroococcidiopsis thermalis PCC 7203	AFY88389.1	KBL
Fischerella spp. PCC 9605	WP_026731944.1	BioF
Gloeothece citriformis	WP_015956773.1	BioF
Hydrococcus spp. RU_2_2	NJM89919.1	BioF
Leptolyngbya spp. 15MV	WP_211740071.1	HemA
Leptolyngbya spp. FACHB-541	WP_190799897.1	BioF
Leptolyngbya spp. PCC 7376	AFY36878.1	KBL
Moorea producens	LYNGBM3L_37440	BioF
Myxacorys almedinensis	WP_162421240.1	BioF
Nostoc cycadae	WP_103126319.1	BioF
Nostoc linckia z16	PHK32241.1	KBL
Nostoc punctiforme	Npun_3567	BioF
Nostoc spp. 3335mG	WP_110154764.1	HemA
Oculatella spp. FACHB-28	WP_190642654.1	BioF
Oscillatoria spp. FACHB-1406	WP_190714779.1	BioF
Phormidesmis priestleyi	WP_068818031.1	BioF
Pleurocapsa spp. PCC 7319	WP_019506998.1	BioF
Rippkaea orientalis	WP_012796615.1	BioF
Scytonema spp. HK-05	WP_073632134.1	BioF
Spirulina spp. SIO3F2	NEO83297.1	BioF
Stanieria cyanosphaera PCC 7437	AFZ37728.1	KBL
Synechocystis spp. PCC 6803	BAA10465.1	BioF
Xenococcus spp. PCC 7305	ELS03286.1	KBL
Bacteroides fragilis	**EYE44679.1 [2]**	**SPT**
Bacteroides fragilis	OCM98910.1	BioF
Bacteroides fragilis	CUA19326.1	KBL
Bacteroides thetaiotaomicron	**SEK37623.1 [3]***	**SPT**
Bacteroides thetaiotaomicron	WP_011107743.1*	KBL
Bacteroides thetaiotaomicron	KXT32562.1*	BioF
Bacteriovorax stolpii	**BAF73753 [4]***	**SPT**
Caulobacter crescentus	**ENZ81852.1 [5]***	**SPT**
Caulobacter crescentus	WP_010919232.1*	HemA
Caulobacter crescentus	KSB87698.1*	BioF
Escherichia coli	EDV60350*	SPT
Escherichia coli	**BAE77675.1 [6]***	**KBL**
Escherichia coli	**1DJ9_A** [7]*****	**BioF**
Gluconobacter oxydans	AAW61792*	SPT
Gluconobacter oxydans	WP_011253160.1*	HemA
Gluconobacter oxydans	WP_011252295.1*	BioF
Granulibacter bethesdensis	AHJ67235.1*	SPT
Granulibacter bethesdensis	WP_025318111.1*	HemA
Granulibacter bethesdensis	AHJ67519.1*	BioF
Nitrosomonas eutropha	PXV80692.1*	SPT
***Porphyromonas gingivalis* str. W83**	**AAQ66781.1 [8]***	**SPT**
Porphyromonas gingivalis	WP_012458311.1*	KBL
Porphyromonas gingivalis	WP_012457897.1*	BioF

Rhodobacter capsulatus	**2BWN_E [9]**	**HemA**
Sphingobacterium multivorum	**BAF73751 [4]***	**SPT**
Sphingomonas paucimobilis	**BAB56013 [10]***	**SPT**
Sphingomonas wittichii	**ABQ70245.1 [11]***	**SPT**
Sphingomonas wittichii	WP_037523345.1*	HemA
Sphingomonas wittichii	WP_011952097.1*	BioF
Zymomonas mobilis	TWD62042.1*	SPT
Zymomonas mobilis	WP_011241021.1*	HemA
Zymomonas mobilis	TWD60409.1*	BioF

Table S11. Marginal amino acid frequencies for the different site classes in the optimal models of SE considered in this study (i.e., LG+FO*H5 and LG+FO+I+G4).

	LG+FO*H5					LG+FO+I+G4
**Amino acid** (proportion)	**Class 1** (18.17%)	**Class 2** (27.14%)	**Class 3** (19.28%)	**Class 4** (17.49%)	**Class 5** (17.92%)	**All classes** (100%)
Ala (A)	0.1258	0.0969	0.1223	0.0676	0.1769	0.1177
Arg (R)	0.0313	0.0261	0.0394	0.0718	0.0726	0.0611
Asn (N)	0.0225	0.0207	0.0225	0.0503	0.0268	0.0318
Asp (D)	0.0279	0.0042	0.0320	0.0775	0.0692	0.0551
Cys (C)	0.0602	0.0387	0.0245	0.0087	0.0038	0.0182
Gln (Q)	0.0048	0.0136	0.0201	0.0421	0.0881	0.0456
Glu (E)	0.0157	0.0001	0.0456	0.0630	0.1189	0.0744
Gly (G)	0.1344	0.1006	0.0373	0.0692	0.0413	0.0571
His (H)	0.0438	0.0123	0.0396	0.0257	0.0264	0.0252
Ile (I)	0.0607	0.1347	0.0414	0.0685	0.0327	0.0633
Leu (L)	0.1030	0.1407	0.1130	0.0913	0.0633	0.0979
Lys (K)	0.0160	0.0129	0.0336	0.0285	0.0733	0.0454
Met (M)	0.0202	0.0216	0.0273	0.0167	0.0220	0.0202
Phe (F)	0.0371	0.0659	0.0672	0.0263	0.0169	0.0376
Pro (P)	0.0489	0.0023	0.0375	0.0928	0.0134	0.0340
Ser (S)	0.0978	0.0950	0.0422	0.0598	0.0463	0.0543
Thr (T)	0.0645	0.0509	0.0950	0.0381	0.0579	0.0560
Trp (W)	0.0001	0.0143	0.0256	0.0086	0.0100	0.0126
Tyr (Y)	0.0003	0.0388	0.0686	0.0269	0.0076	0.0302
Val (V)	0.0849	0.1098	0.0653	0.0663	0.0326	0.0626

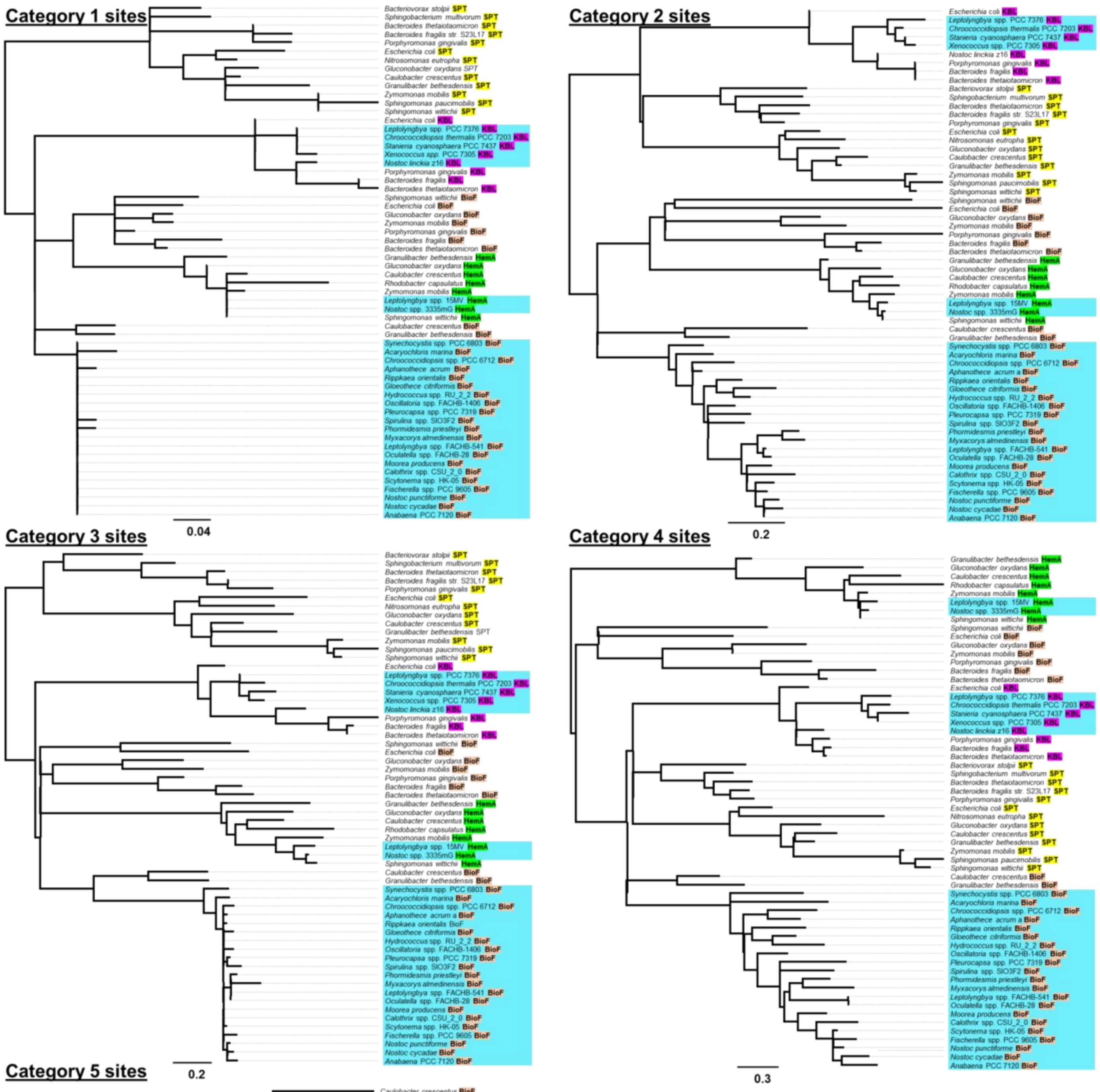

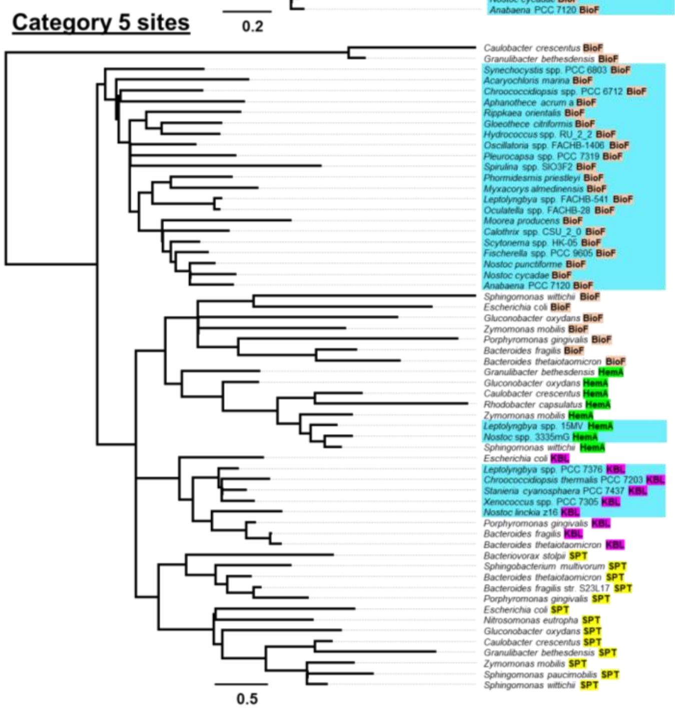

Fig. S13. Unrooted phylogeny of the AOS-family of enzymes, inferred using maximum likelihood under the LG+FO*H5 model of SE, with the edge lengths drawn to scale for the five classes (categories) of sites in the H5 model. The bar below each sub-figure represents substitutions per site for the corresponding category of sites. The sums of edge lengths for the four categories of sites are 2.6, 13.9, 19.22, 29.5 and 56.6, so the four classes of sites have clearly evolved at different rates.

1. **Geiger O, González-Silva N, López-Lara IM, Sohlenkamp C**. Amino acid-containing membrane lipids in bacteria. *Prog Lipid Res* 2010;49:46–60.

2. **An D, Na C, Bielawski J, Hannun YA, Kasper DL**. Membrane sphingolipids as essential molecular signals for Bacteroides survival in the intestine. *Proc Natl Acad Sci U S A* 2011;108 Suppl 1:4666–71.

3. **Johnson EL, Heaver SL, Waters JL, Kim BI, Bretin A, *et al.*** Sphingolipids produced by gut bacteria enter host metabolic pathways impacting ceramide levels. *Nat Commun* 2020;11:2471.

4. **Ikushiro H, Islam MM, Tojo H, Hayashi H**. Molecular characterization of membrane-associated soluble serine palmitoyltransferases from Sphingobacterium multivorum and Bdellovibrio stolpii. *J Bacteriol* 2007;189:5749–61.

5. **Stankeviciute G, Guan Z, Goldfine H, Klein EA**. Caulobacter crescentus Adapts to Phosphate Starvation by Synthesizing Anionic Glycoglycerolipids and a Novel Glycosphingolipid. *mBio*;10:e00107-19.

6. **Schmidt A, Sivaraman J, Li Y, Larocque R, Barbosa JA, *et al.*** Three-dimensional structure of 2-amino-3-ketobutyrate CoA ligase from Escherichia coli complexed with a PLP-substrate intermediate: inferred reaction mechanism. *Biochemistry* 2001;40:5151–5160.

7. **Alexeev D, Alexeeva M, Baxter RL, Campopiano DJ, Webster SP, *et al.*** The crystal structure of 8-amino-7-oxononanoate synthase: a bacterial PLP-dependent, acyl-CoA-condensing enzyme. *J Mol Biol* 1998;284:401–419.

8. **Moye ZD, Valiuskyte K, Dewhirst FE, Nichols FC, Davey ME**. Synthesis of Sphingolipids Impacts Survival of Porphyromonas gingivalis and the Presentation of Surface Polysaccharides. *Front Microbiol* 2016;7:1919.

9. **Astner I, Schulze JO, van den Heuvel J, Jahn D, Schubert W-D, *et al.*** Crystal structure of 5-aminolevulinate synthase, the first enzyme of heme biosynthesis, and its link to XLSA in humans. *EMBO J* 2005;24:3166–3177.

10. **Ikushiro H, Hayashi H, Kagamiyama H**. A water-soluble homodimeric serine palmitoyltransferase from Sphingomonas paucimobilis EY2395T strain. Purification, characterization, cloning, and overproduction. *J Biol Chem* 2001;276:18249–56.

11. **Raman MC, Johnson KA, Clarke DJ, Naismith JH, Campopiano DJ**. The serine palmitoyltransferase from Sphingomonas wittichii RW1: An interesting link to an unusual acyl carrier protein. *Biopolymers* 2010;93:811–22.
